# Unraveling the chicken T cell repertoire with enhanced genome annotation

**DOI:** 10.3389/fimmu.2024.1359169

**Published:** 2024-03-14

**Authors:** Simon P. Früh, Martin A. Früh, Benedikt B. Kaufer, Thomas W. Göbel

**Affiliations:** ^1^ Department of Veterinary Sciences, Ludwig-Maximilians-Universität München, Munich, Germany; ^2^ Institute of Virology, Freie Universität Berlin, Berlin, Germany; ^3^ Independent Researcher, Munich, Germany

**Keywords:** chicken, T cells, TCR α/β/γ/δ locus annotation, *VJ-gene-finder*, TCR repertoire sequencing, spleen

## Abstract

T cell receptor (TCR) repertoire sequencing has emerged as a powerful tool for understanding the diversity and functionality of T cells within the host immune system. Yet, the chicken TCR repertoire remains poorly understood due to incomplete genome annotation of the TCR loci, despite the importance of chickens in agriculture and as an immunological model. Here, we addressed this critical issue by employing 5’ rapid amplification of complementary DNA ends (5’RACE) TCR repertoire sequencing with molecular barcoding of complementary DNA (cDNA) molecules. Simultaneously, we enhanced the genome annotation of TCR Variable (V), Diversity (D, only present in β and δ loci) and Joining (J) genes in the chicken genome. To enhance the efficiency of TCR annotations, we developed *VJ-gene-finder*, an algorithm designed to extract VJ gene candidates from deoxyribonucleic acid (DNA) sequences. Using this tool, we achieved a comprehensive annotation of all known chicken TCR loci, including the α/δ locus on chromosome 27. Evolutionary analysis revealed that each locus evolved separately by duplication of long homology units. To define the baseline TCR diversity in healthy chickens and to demonstrate the feasibility of the approach, we characterized the splenic α/β/γ/δ TCR repertoire. Analysis of the repertoires revealed preferential usage of specific V and J combinations in all chains, while the overall features were characteristic of unbiased repertoires. We observed moderate levels of shared complementarity-determining region 3 (CDR3) clonotypes among individual birds within the α and γ chain repertoires, including the most frequently occurring clonotypes. However, the β and δ repertoires were predominantly unique to each bird. Taken together, our TCR repertoire analysis allowed us to decipher the composition, diversity, and functionality of T cells in chickens. This work not only represents a significant step towards understanding avian T cell biology, but will also shed light on host-pathogen interactions, vaccine development, and the evolutionary history of avian immunology.

## 1 Introduction

The avian immune system plays a pivotal role in safeguarding poultry health, thereby contributing significantly to human food security. Among the various components of the avian immune system, T cells are instrumental in orchestrating adaptive immune responses. Cytotoxic cluster of differentiation (CD)8^+^ T cells target infected or aberrant host cells, whereas CD4^+^ T helper cells facilitate B cell functions and coordinate effector cells and molecules. In addition to αβ T cells, γδ T cells likely also play important roles in homeostasis and infection, but their functions are less well characterized and appear to comprise both innate and adaptive effector functions (1–3). Monoclonal antibodies specific for major chicken T cell antigens CD3, CD4, CD8, TCR γδ (clone TCR-1), TCR αβ Vβ1 (clone TCR-2), TCR αβ Vβ2 (clone TCR-3) have been available for more than 30 years (4–9). Yet, a detailed understanding of the intricate dynamics of T cell responses in chickens remains elusive. Unraveling the complexities of T cell-mediated immunity requires analysis of T cells on the clonal level.

Clonal T cell populations express identical TCRs on their surface, leading to shared antigen-specificity. The TCR structure comprises either an α and a β chain, or a γ with a δ chain, in heterodimeric form (8, 10). During thymic development, naïve T cells are generated, each possessing a distinct specificity in their TCR. This specificity arises from somatic DNA recombination of V(D)J genes, where the resulting variable domains are spliced to the Constant (C) domain exons at the 3’ end and to the first exon (L-PART1; nomenclature according to IMGT, the international Immunogenetics database) of the signal (leader) peptide at the 5’ end (11, 12). Notably, the 5’ leader peptide in chicken Vα1 genes is uniquely encoded within a single exon (13, 14). Somatic DNA recombination occurs separately for each chain and is essential for generating a vast diversity of antigen receptors. The process is initiated by the recombination activating genes (RAG) recombinase that binds to conserved recombination signal sequences (RSSs) flanking each V, D and J gene segment. RSSs contain conserved heptamers and nonamers separated by a 12- ( ± 1) or a 23-mer ( ± 1) spacer, governing V(D)J recombination according to the 12-23 rule (11, 12). The highly variable CDR3 at the V(D)J junction primarily interacts with the target peptide (10, 15).

Previous work has identified V(D)J and C genes in the chicken genome that are arranged in highly structured clusters: The TCR β locus (TRB) spans a region of approximately 210 kilobases (kb) on chromosome 1 (16–20), the TCR γ locus (TRG) spans a region of approximately 82 kb on chromosome 2 (19, 21–23), and TCR α (TRA) and TCR δ (TRD) genes are arranged in a hybrid locus of approximately 800 kb on chromosome 27, with the TCR δ sequences nested between the Vα and Jα genes (13, 14, 19, 24). In addition, a separate small TCR δ-like locus was identified on chromosome 10 comprised of a single cassette with one copy of immunoglobulin heavy chain (IgH) V-like VHδ, Dδ, Jδ and Cδ genes (25). V(D)J genes recognized within each locus were organized into subfamilies, and classified based on their predicted functionality as either functional (F) genes, open reading frame (ORF) genes or pseudogenes (P) (26). Unfortunately, however, annotation efforts as part of different studies focusing on the same locus reached varying conclusions regarding the number of VJ genes and V families. The observed incongruity likely stems from inconsistent gene annotation methodologies and variations in the genomes utilized. Furthermore, the challenge of comparability has been exacerbated by instances where the annotated sequences were not universally accessible in the public domain (14, 19). Recent studies have provided robust annotations of the TCR β and TCR γ loci (20, 22, 23). However, comprehensive details regarding the TCR α/δ sequences are still unavailable.

In recent years, TCR repertoire sequencing has been widely used in mammals to dissect the intricacies of T cell-mediated immunity. TCR profiling enables the characterization of T cell diversity at baseline and facilitates quantification of clonal expansion and memory recall responses to microbial challenges (27, 28). Current research endeavors have expanded this approach to chicken samples, offering a comprehensive analysis of private and public β and γ T cell populations across various tissues and microbial conditions (22, 23, 29). However, current knowledge about chicken α and δ chain sequences remains incomplete.

In this study, we set to address gaps in the annotation of *Gallus gallus* TCR loci and expanded upon previous investigations involving chicken TCR repertoires. Molecular barcoding of cDNA molecules with unique molecular identifiers (UMIs) was integrated in our pipeline, thereby enhancing the quantitative precision essential for comprehensive TCR repertoire analysis (30, 31). We provide a comprehensive annotation of all TCR loci within the Huxu chicken genome and a TCR profiling pipeline with baseline data on the physiologic TCR α, β, γ and δ repertoires in the spleen. We developed a tool designed to streamline the annotation of chicken TCR genes in novel genome assemblies termed *VJ-gene-finder*, that was made accessible to the public along with all sequence data. This will provide the basis for new investigations into T cell-mediated immunity in chickens.

## 2 Materials and methods

### 2.1 Animals

White Leghorn line M11 chickens (Friedrich-Loeffler-Institute, Federal Research Institute for Animal Health, Neustadt, Germany) were hatched and conventionally housed with ad libitum access to water and a commercial diet. Chickens were euthanized for tissue collection at 9 - 13 weeks of age. Splenic tissue was collected and stored in RNAlater (Sigma-Aldrich, Burlington, MA, USA) immediately post-mortem, then incubated for 24 hours at 4°C followed by long-term storage at -20°C.

### 2.2 RNA

Ribonucleic acid (RNA) was extracted using the SV Total RNA Isolation System (Promega, Madison, WI, USA) with an on-column incubation with deoxyribonuclease I (DNase I), quantified on a Nanodrop ND-1000 (Thermo Fisher Scientific, Waltham, MA, USA) and RNA quality was determined on a 2100 Bioanalyzer (RNA Integrity Number > 9) (Agilent Technologies, Santa Clara, CA, USA).

### 2.3 Primer design for chicken TCR chains

For each TCR chain, an outer reverse 1 (*R1*) gene-specific primer for 5’ RACE and two nested primers (*R2* and *R3*) were designed to bind to C exons of α (GenBank EF554736), β (GenBank EF554782), γ (GenBank NM_001318455) or δ (GenBank AF175433) chains using Geneious Prime 2022.0.1 (https://www.geneious.com/). The *R3* for each chain was designed to bind close to the 5’ end of the C gene. Target-specificity was confirmed by NCBI Primer-BLAST against the *Gallus gallus* RefSeq messenger RNA (mRNA) database (32). Primer sequences are listed in **Table 1**.

**Table 1:** Primer sequences for TCR-specific 5’ RACE and PCR amplification.

Step	Name	Sequence	F/R	Chain
cDNA	SmartNNNNa*	*AAGCAGUGGTAUCAACGCAGAGUNNNNUNNNNUNNNNUCTTrGrGrGrG*	F	αβγδ
cDNA	*chTRAC_R1*	*CTGTCTTACTATCGACTGAG*	R	α
cDNA	*chTRBC_R1*	*ACCTTCCAGACTAAATTGAG*	R	β
cDNA	*chTRGC_R1*	*CATCGGTCCATTTCACCCGA*	R	γ
cDNA	*chTRDC_R1*	*TCATTAGAGGACATCTCCAAA*	R	δ
PCR1	Smart20*	*CACTCTATCCGACAAGCAGTGGTATCAACGCAG*	F	αβγδ
PCR1	*chTRAC_R2*	*GGTCAGCCTGTAGACTGAAGG*	R	α
PCR1	*chTRBC_R2*	*TGCTTTGATGGTGAAAAGATGACC*	R	β
PCR1	*chTRGC_R2*	*TCATGTTCCTCCTGCATGATTTC*	R	γ
PCR1	*chTRDC_R2*	*TGATTTCATCACAATGACCTCTGG*	R	δ
PCR2	Step_1*	*(acactctttccctacacgacgctcttccgatct)XXXXXCACTCTATCCGACAAGCAGT*	F	αβγδ
PCR2	*chTRAC_R3*	*(gactggagttcagacgtgtgctcttccgatct)XXXXXGTAGACTGAAGGAGATGGAGTAAT*	R	α
PCR2	*chTRBC_R3*	*(gactggagttcagacgtgtgctcttccgatct)XXXXXGGTTCTATGATTTCACTGTTCTTCC*	R	β
PCR2	*chTRGC_R3*	*(gactggagttcagacgtgtgctcttccgatct)XXXXXCTGGTGCTGAACTTCCTTTGTC*	R	γ
PCR2	*chTRDC_R3*	*(gactggagttcagacgtgtgctcttccgatct)XXXXXGAATAGAATCTCTCTGCTCCC*	R	δ

*Adapted from (31); N = any nucleotide (*A, T, G or C*); *rG* = riboguanosine; *U* = deoxyuracil; () = overhang for library preparation; *XXXXX* = optional sample barcode; F = forward primer; R = reverse primer.

### 2.4 VJ-gene-finder

A new search algorithm “*VJ-gene-finder”* was developed to identify and extract functional V and J genes from the chicken genome, based on characteristic biological patterns that define immunoglobulin V and J genes in many species (11, 33). The features used include conserved amino acid residues at specific positions (for V segments according to IMGT nomenclature: “1^st^-CYS”, “CONSERVED-TRP” and “YYC/YFC/YLC/YHC/YIC/TFC” motif that includes the “2^nd^-CYS”; for J segments: “FG” motif), conserved nucleic acid motifs in genes and at specific positions (for V genes with a single-exon leader sequence: *ATG* start codon, for V genes with a spliced leader sequence: splice acceptor sequence *AG*; for J segments: *TTYGGNNNNGG* and *TNNBNRT*, and splice donor sequence *GTRDGD*) and conserved recombination signal sequences (for both V and J segments: begin with *CAC* nucleic acid motif) in combination with length constraints and the requirement for an open reading frame with or without splicing. A summary of the algorithm for V genes (**Supplementary Figures 1A, B**) and J genes (**Supplementary Figures 2A, B**) is included in the **Supplementary Material**. *VJ-gene-finder* was written in Python and it was made publicly available as a free and open-source software (https://github.com/simonfrueh/VJ-gene-finder). The algorithm is similar to the method used by Oliveri et al. (33), but was modified to enable identification of chicken TCR V genes that are encoded by a single exon (together with the leader sequences) (13) and an additional function was added to search and extract J gene candidates. Additionally, *VJ-gene-finder* tentatively assigns candidate V genes to chicken V gene families based on amino acid motifs near the 5’end (TRAV1: “QVQQ”, TRAV2: “VSQQ”, TRAV3: “LQYP”, TRBV1: “LQQT”, TRBV2: “EINQ”, TRBV3: “ITQW, TRGV1: “QVLLQQ”, TRGV2: “PIQS”, TRGV3: “QAVPMQ” or “QAAPVQ, TRGV4: “LWQSP”, TRDV1: “ETSGGGV”, TRDV2: “LEASGGG”, “TRDV3: “VEFGGDV”, TRDV4: “RIVEAG”, TRDV5: “EIHAKKSA”, TRDVH1: “QIEMVTT”).

### 2.5 Annotation of TCR loci

*VJ-gene-finder* (v0.1) was used to identify putative V and J gene sequences that match the search criteria in chromosomes 1, 2, 27 and 10 of the Huxu chicken genome assembly GGswu (GenBank assembly GCA_024206055.2) (34). The search criteria were not specific to VJ segments, thus the algorithm also extracted non-VJ segments outside of the TCR loci. *Clustal* multiple sequence alignment of candidate sequences in Jalview revealed clusters of highly similar (functional) V and J genes (**Supplementary Figures 1C, 2C**) (35–37). The chromosomal location of these genes defined the TCR locus for each TCR chain. *VJ-gene-finder* hits outside of the TCR locus were more dissimilar to each other and were discarded (**Supplementary Figures 1C, 2C**). Each putative V gene was manually annotated in Artemis Release 18.2.0 (38) and examined for the presence of predicted functional features, including a start codon, an RSS, leader sequences and splice sites using *Recombination Signal Sequences Site*, *SignalP 6.0* and *Spliceator* (**Supplementary Figure 1D**) (39–41). J genes were manually annotated for the presence of a functional RSS and splice site using the same tools (**Supplementary Figure 2D**). By design, *VJ-gene-finder* only identified functional (F) and open reading frame (ORF) V and J genes (IMGT functionality nomenclature) (26). To identify V and J pseudogenes (P), potentially unidentified VJ F and ORF genes, and to detect D genes, TCR amplicon sequences were aligned to the Huxu chromosomes. These amplicons were generated in our laboratory as part of a different study from the same chicken line, following the same amplification strategy, with RNA from fluorescence-activated cell sorting (FACS)-isolated peripheral blood T cells. To enable partial and local alignment of spliced and rearranged TCR sequences to the un-rearranged genome, we employed the *bowtie2* (v2.3.5.1) aligner with options *–no-unal –local* (42). Raw reads were directly aligned to the reference sequence, converted to bam files, sorted and indexed using *samtools* (v1.15.1) and the alignment was visualized in *Artemis* (43). Regions with aligned partial TCR sequences were examined for the presence or absence of RSSs, splice sites, conserved amino acid and conserved nucleotide sequences as described above.

V genes were classified as functional when all conserved amino acids were identified, when a predicted 23-mer spacer (23)RSS was found at the 3’ end and the (spliced) leader sequence at the 5’ end encoded a predicted signal peptide, and frameshift mutations or stop codons were absent. The length of the V gene was defined ranging from the cleavage site of the signal peptide as predicted by *SignalP 6.0* to the beginning of the RSS (*CAC* motif) (**Supplementary Figures 1A, B, D**). J genes were classified as functional only when stop codons were absent and when the conserved “FG” motif, a predicted 12-mer spacer (12)RSS at the 5’end and a splice donor at the 3’ end were identified (**Supplementary Figures 2A, B, D**). The RSS and splice site also defined the beginning and end of the J gene. D genes were identified using *bowtie2* alignments and were defined as a sequence between a 12RSS and a 23RSS without any stop codons. Sequences without stop codons or frameshift mutations that were altered in one of the above-mentioned features were classified as ORFs. Sequences with aligned reads that contained stop codons, frameshift mutations or an RSS without *CAC* motif were assigned pseudogenes. Pseudogenes and the length of pseudogenes could not always be identified unambiguously because pseudogenes, by definition, lack certain characteristics that define immunoglobulin genes. All identified V(D)J genes and alterations (if applicable) are summarized in **Supplementary Table 1**.

### 2.6 V family assignment, V and J gene numbering, CDR and FR annotation

TCR V genes with ≥75% nucleotide sequence identity were grouped into V families following the nomenclature outlined by the international ImMunoGeneTics information system nomenclature (IMGT) (11). For V family assignment, DNA sequences of V genes were analyzed using EMBL-EBI online analysis tools (44). For each chain, V genes were aligned using Clustal Omega, clade consensus sequences were determined with *EMBOSS Cons* and percent identity to the group consensus was calculated with *MView* (45). The identity threshold for V gene families was 75%, except for TRGV2-27 that was assigned to the TRGV2 family with an identity of 73.8% compared to the group consensus. Chicken V families were assigned numbers based on established nomenclature from prior studies, where applicable (20, 23). Additionally, new V families, including those within the δ locus, as well as all V genes within each family, were numbered in ascending order from 5’ to 3’ (towards the C gene). Consistent with previous conventions in chickens, J genes were similarly numbered sequentially in ascending order from 5’ to 3’ direction, progressing towards the C gene (20, 22, 23). CDR and framework (FR) regions were defined by alignment with *IMGT/DomainGapAlign* against Homo sapiens V Domain reference sequences (46). TRAJ genes were annotated based on the conserved “F/WG.G” motif. The reading frame of unconventional TRAJ genes that lacked the motif was examined in several individual sequencing reads and anchor points “CDR3 end” and “FR4 begin” were defined accordingly.

### 2.7 Comparison to previous TCR gene annotations

Only full-length V genes > 222 bp among the genes annotated in this study were considered for the sequence comparison. For each locus, reference sequences from previous studies were queried against the references sequences from this study by pairwise alignment with *Biopython* (v1.83) using *pairwise2.align.globalxx()* (47). Alignments were compared using the following formulas: $percent\;identity\;=\frac{identical\;positions}{length\;of\;shorter\;sequence}\;\times 100\;$ and the number of mismatches =length of shorter sequence − identical positions . The best match with the highest percent identity, or multiple matches in case of equal percent identity, were reported for each query sequence.

### 2.8 Evolutionary analysis

Evolutionary analysis were conducted in *MEGA11* as previously described (48, 49). In brief, all VJ (functional and ORF) DNA sequences annotated as part of these studies were aligned using *MUSCLE*. The best substitution model was identified by *MEGA11* (K2+G+I) and used to construct a Maximum Likelihood Tree (with partial deletion option). The reliability of the tree was estimated with 500 bootstrap replicates and the tree was visualized using *iTOL v6* (50). Dotplots were created with *Unipro UGENE* (v48.1) with a minimum repeat length of 100 bp and a repeat identity threshold of 100% (51). Locus representation plots were created with *DNA Features Viewer* (v3.1.3) (52).

### 2.9 5’ RACE

A previously described approach for TCR-specific 5’ RACE was modified for chicken samples (31, 53). A UMI was included in the template switching oligo for *in silico* removal of polymerase chain reaction (PCR) duplicates and improved error correction (30). In addition, 5-nucleotide sample barcodes were added at the 5’ end to the primers of the second PCR (chain-specific) for demultiplexing of pooled samples (**Table 1**). Depending on the sequencing provider and the library preparation method, specific adapters sequences can also be added to the primers of the second PCR (31, 54). In this study, the PCR 2 oligonucleotides contained the following adapter sequences: *ACACTCTTTCCCTACACGACGCTCTTCCGATCT* (forward primer) and *GACTGGAGTTCAGACGTGTGCTCTTCCGATCT* (reverse primer). For first strand cDNA synthesis with template switching, 500 ng of purified total RNA in 3 μl nuclease-free water were combined with 1.5 μl antisense primer mix containing equal amounts of 10 μM reverse primers (*chTRAC_R1* and *chTRBC_R1* for amplification of αβ TCRs or *chTRGC_R1* and *chTRDC_R1* for amplification of γδ TCRs). The template-antisense primer mixture was incubated at 72°C for 3 minutes and at 42°C for 2 minutes. Then 5.5 μl of reverse transcription master mix containing 2 μl of 5x First-Strand Buffer, 0.25 μl of 100 mM DL-Dithiothreitol, 1 μl of deoxynucleotide triphosphates (dNTPs) (10 mM each) (both Promega, Madison, WI, USA), 1 μl of 10 μM *SmartNNNNa* primer, 0.25 μl of ribonuclease (RNase) Inhibitor (40 U/μl) and 1 μl SMARTScribe Reverse Transcriptase (both Takara Bio, Kusatsu, Shiga, Japan) was added to each sample and the first strand synthesized by incubation at 42°C for 90 minutes and at 70°C for 10 minutes. The cDNA was cooled on ice, then 5 μl USER Enzyme (1 U/μl) (New England Biolabs, Ipswich, MA, USA) was added and the mixture was incubated at 37°C for 60 minutes. cDNA was stored at -20°C.

### 2.10 Polymerase chain reaction and amplicon next generation sequencing

For the first PCR (PCR 1), 5 μl of template cDNA was combined with 35 μl nuclease-free water, 5 μl 10x Advantage 2 PCR Buffer, 1 μl dNTPs (10 mM each) (Promega, Madison, WI, USA), 1 μl Smart20 primer (10 μM), 2 μl antisense primer mix containing equal amounts of 10 μM reverse primers (*chTRAC_R2* and *chTRBC_R2* for amplification of αβ TCRs or *chTRGC_R2* and *chTRDC_R2* for amplification of γδ TCRs) and 1 μl 50x Advantage 2 Polymerase Mix (Takara Bio, Kusatsu, Shiga, Japan). PCR 1 was carried out by incubation at 95°C for 1 minute, followed by 19 repeated cycles of incubation at 95°C for 20 seconds (s), 65°C for 20 s and 68°C for 50 s, followed by a final extension at 68°C for 3 minutes. PCR 1 amplicons were purified with AMPure XP beads (Beckman Coulter, Brea, CA, USA) using a ratio of PCR 1 Reaction Volume (μl): AMPure XP Volume (μl) of 1: 0.65, with two washes with 80% Ethanol on a SMARTer-Seq Magnetic Separator (Takara Bio, Kusatsu, Shiga, Japan) and elution in 27 μl nuclease-free water. The second PCR (PCR 2) was carried out separately for each TCR chain. The reaction mix contained 19.5 μl nuclease-free water, 2.5 μl 10x Advantage 2 PCR Buffer, 0.5 μl dNTPs (10 mM each), 0.5 μl *Step_1* primer (10 μM), 0.5 μl *R3* chain-specific reverse primer (10 μM), 0.5 μl 50x Advantage 2 Polymerase Mix with 1 μl purified PCR 1 product as template. For δ chains, 2 μl purified PCR 1 product was used as template. The PCR 2 cycling conditions were the same as for PCR 1, except with a different number of repeated cycles (13 cycles for α, 11 cycles for β, 15 cycles for γ and 15 cycles for δ). PCR 2 amplicons were purified by gel electrophoresis on a 1.25% Agarose gel with Novel Juice DNA stain (Sigma-Aldrich, Burlington, MA, USA), excision of bands of the desired size [~600 - 650 base pairs (bp)] on a blue light table and gel extraction with the Monarch DNA Gel Extraction Kit (New England Biolabs, Ipswich, MA, USA). Of note, in some cases (particularly with α and γ chain amplicons), secondary bands ~100 - 200 bp larger than the desired size were observed, which were likely cDNA molecules with long UTRs and/or incomplete or false splicing. Those extra bands were excluded during gel extraction. Purified PCR 2 amplicons were quantified with a Quantus Fluorometer and the QuantiFluor dsDNA System (both Promega, Madison, WI, USA). Library preparation (2^nd^ PCR Amplicon option) and paired-end sequencing at 2x300 bp on an Illumina MiSeq Instrument (Illumina, San Diego, USA) was performed by Eurofins Genomics Germany GmbH (Ebersberg, Germany).

### 2.11 Quality control and TCR sequence analysis

The quality of raw reads was determined using *fastqc* (v0.11.9) and *MultiQC* (v1.15) (55, 56) and reads were analyzed with *MiXCR* (v4.2.0) ( (57). A custom germline V(D)J library containing chicken α, β, γ and δ chain sequences was created with *repseqio* (v1.3.5) [now part of *MiXCR* (v4)]. In brief, all functional and ORF segments (annotated in this study) were exported as Fasta files and converted into a JSON structured library with *repseqio* using the *fromPaddedFasta* option. Anchor points were specified manually for V genes *(-FR1Begin, -CDR1Begin, -FR2Begin, -CDR2Begin, -FR3Begin, -CDR3Begin, -VEnd*), D genes (*-DBegin, -DEnd)* and J genes (*-JBegin, -FR4Begin, -FR4End*) and annotated sequences were compiled into one chicken VDJ library with *repseqio merge*. This library was used in *MiXCR* to align and annotate TCR sequences. Alignment (*mixcr align*) was used with options *-p generic-tcr-amplicon-umi*, *-OallowChimeras=true, –tag-parse-unstranded*, *–rna*, *–rigid-left-alignment-boundary*, *–floating-right-alignment-boundary C*, –tag pattern ‘^*(UMI : TNNNNTNNNNTNNNNT)ctt(R1:*)\^(R2:*)’. Tags were refined with *mixcr refineTagsAndSort*, CDR3 clonotypes were assembled with *mixcr assemble* and clonotype tables exported with *mixcr exportClones*. Clonotype tables were randomly downsampled within each chain to the weighted total number of clonotypes of the smallest sample and analyzed and visualized using *Jupyter Notebook* (v7.0.6) with *python* (v3.11.7), *matplotlib* (v3.8.2), *numpy* (v1.26.2), *pandas* (v2.1.3), *scipy* (v1.11.4) and *seaborn* (v0.13.0) (58–62). Additional analyses were conducted using *R* software (v4.3.2) with *resphape2* (v1.4.4) and *tidyverse* packages (v2.0.0) (63, 64).

### 2.12 Statistical analysis

Statistical analyses were conducted using *R* software (v4.3.2). A negative binomial generalized linear model (GLM) was fitted to account for overdispersion, with counts of T cell receptor sequences or counts of amino acids in T cell receptor sequences as the response variable and V gene type, V family type, J gene type, or amino acid type as predictors. This analysis was performed using the *glm.nb()* function from the *MASS* package (v7.3-60) (65). The overall significance of predictors in the model was assessed through analysis of deviance tests using the *Anova()* function from the *car* package (v3.1-2) (66). For *post-hoc* pairwise comparisons among levels of the predictors, the *emmeans* package (v1.10.0) was employed, applying Tukey adjustment for multiple comparisons (67).

## 3 Results

### 3.1 The chicken TCR loci

**Figure 1:**
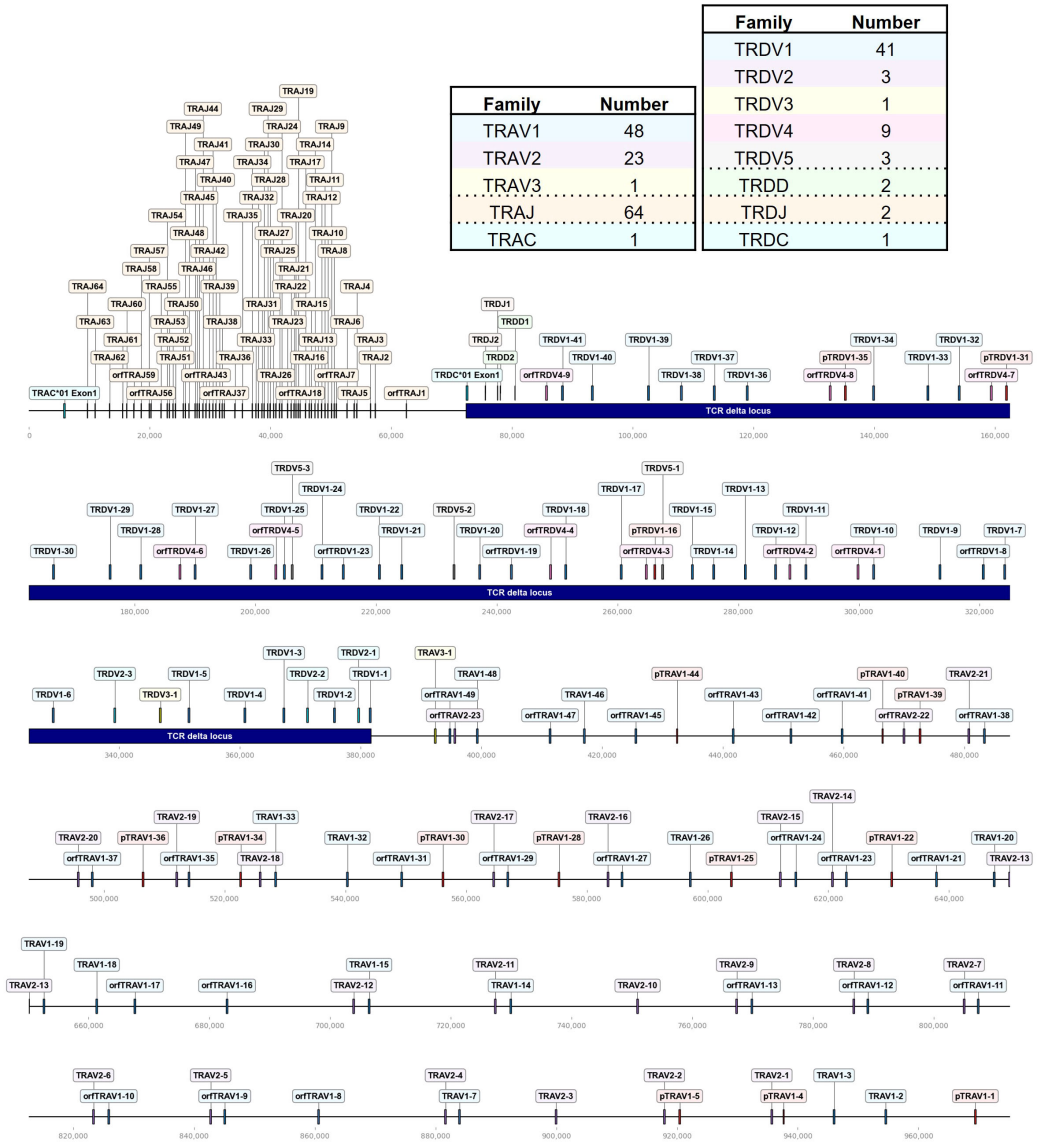
The chicken TCR α/δ locus on chromosome 27. To-scale representation of the TCR α/δ hybrid locus within the reverse strand on chromosome 27 (Huxu chicken genome) with V(D)JC genes labeled, and summary tables with total gene counts. The DNA segment presented was specifically chosen to cover the entire TCR locus. Gene names composed of chain (TRA = TCR α, TRD = TCR δ), type (V, variable; D, diversity; J, joining), family, and an individual number (in ascending order) separated by a dash. “p” prefix indicates pseudogenes and “orf” prefix indicates open reading frames. The region containing the TCR δ locus was indicated by a blue bar. Only the first exon of the constant (C) gene was depicted.

**Figure 2:**
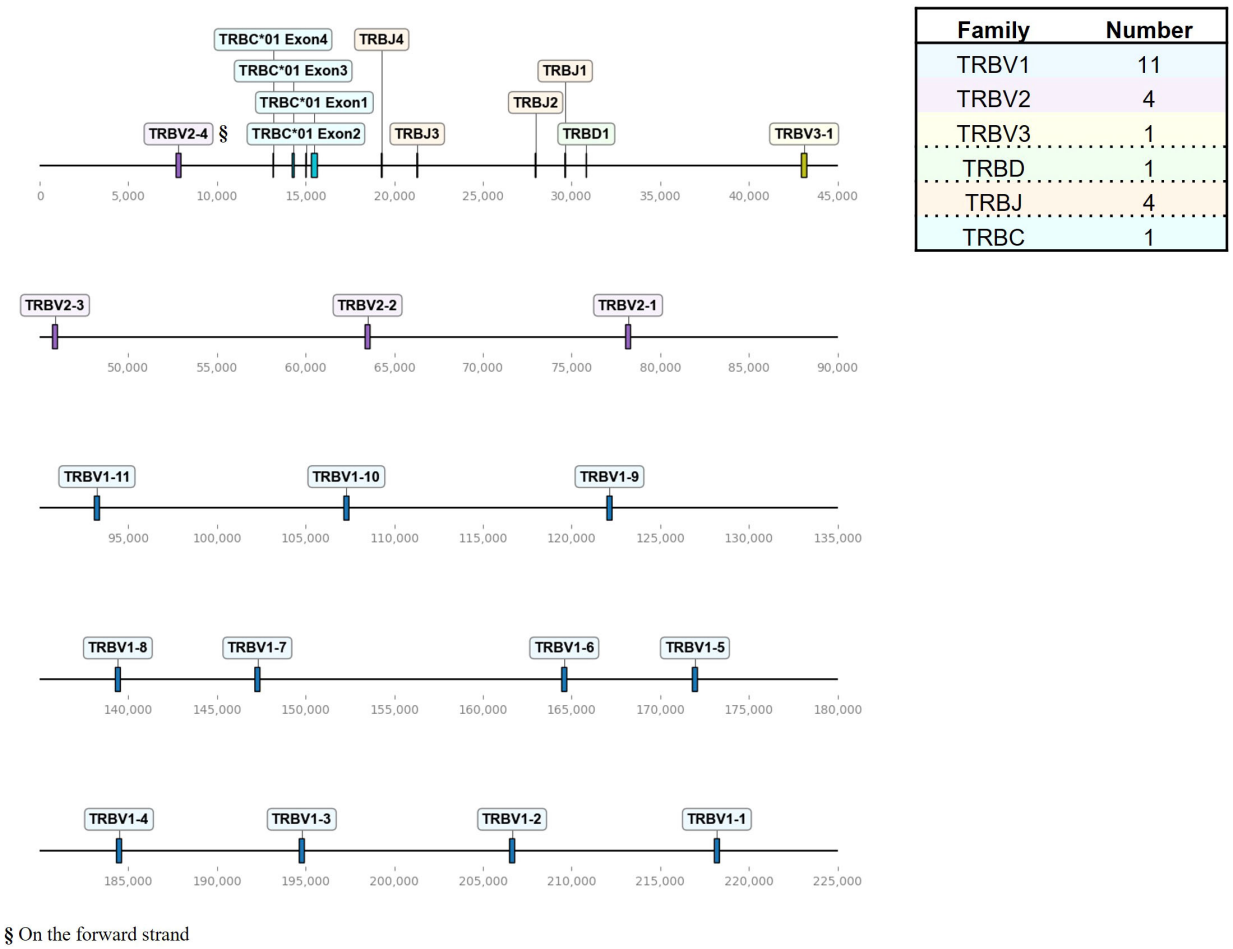
The chicken TCR β locus on chromosome 1. To-scale representation of the TCR β locus within the reverse strand on chromosome 1 (Huxu chicken genome) with VDJC genes labeled, and a summary table with total gene counts. The DNA segment presented was specifically chosen to cover the entire TCR locus. Gene names composed of chain (TRB = TCR β), type (V, variable; D, diversity; J, joining), family, and an individual number (in ascending order) separated by a dash. “p” prefix indicates pseudogenes and “orf” prefix indicates open reading frames.

**Figure 3:**
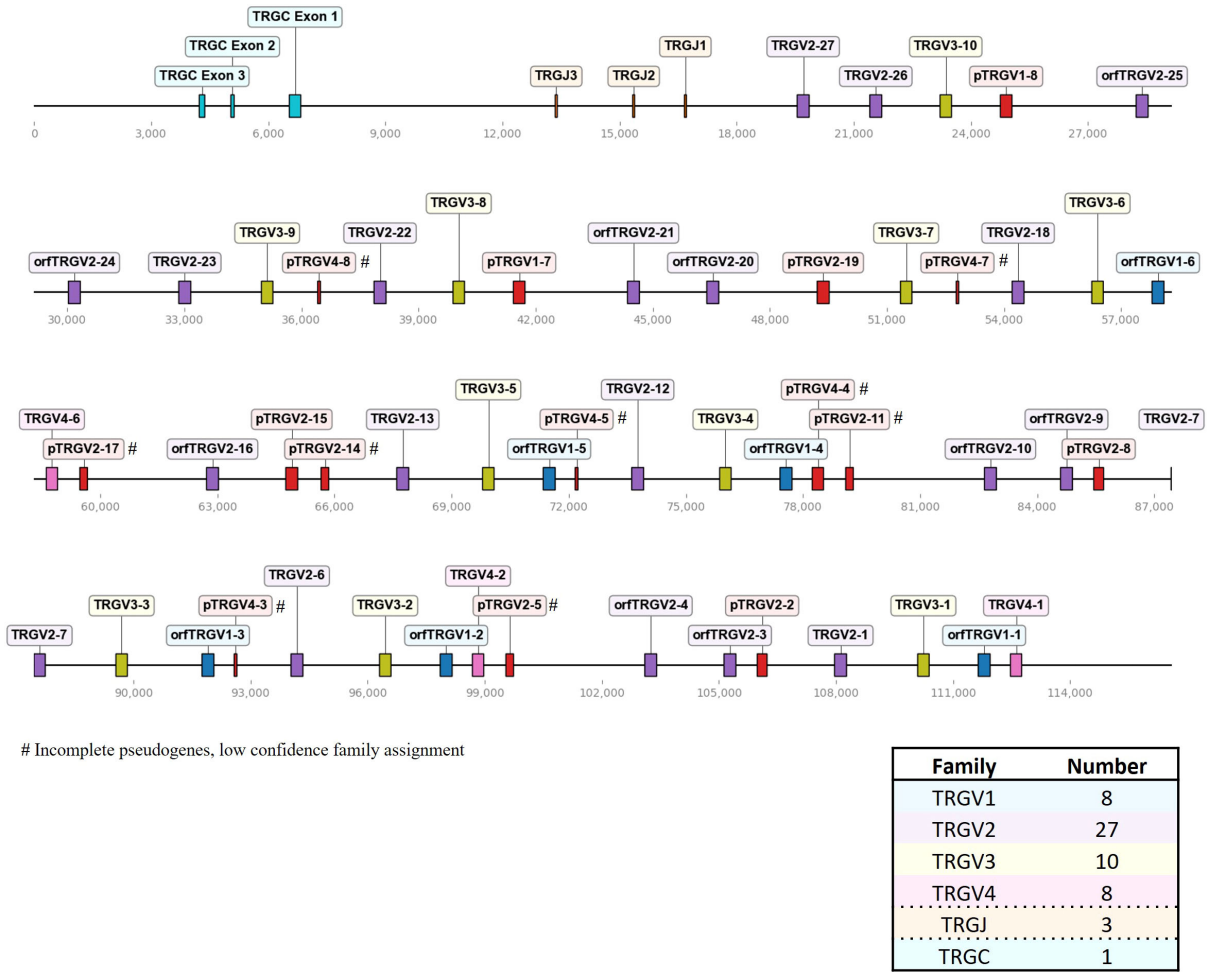
The chicken TCR γ locus on chromosome 2. To-scale representation of the TCR γ locus within the reverse strand on chromosome 2 (Huxu chicken genome) with VJC genes labeled, and a summary table with total gene counts. The DNA segment presented was specifically chosen to cover the entire TCR locus. Gene names composed of chain (TRG = TCR γ), type (V, variable; D, diversity; J, joining), family, and an individual number (in ascending order) separated by a dash. “p” prefix indicates pseudogenes and “orf” prefix indicates open reading frames.

Our goal was to establish a targeted TCR repertoire analysis for all four chicken TCR chains using Next Generation Sequencing (NGS). Bioinformatic analysis of expressed TCRs relies upon a comprehensive annotation of the germline V(D)J repertoire, enabling identification of functional regions (FRs, CDRs) and germline gene identity. To generate an updated and systematic annotation of the chicken TCR loci, we semi-automatically extracted VJ genes from TCR loci within the recent Huxu chicken genome assembly (34) using *VJ-gene-finder*, a program that was generated as part of this study. The search algorithm of *VJ-gene-finder* is based on the method by Olivieri et al., with chicken-specific adjustments to the motif criteria and new functionality for extraction of putative J genes and V genes with a single-exon leader peptide (**Supplementary Figures 1A, B, 2A, B**) (33). *VJ-gene-finder* hits were manually curated and functional RSSs, splice sites and signal peptides were verified (**Supplementary Figures 1C, D, 2C, D**). Pseudogenes and additional genes, not identified by *VJ-gene-finder*’s search parameters, were manually annotated. This annotation process was facilitated by the local (partial) alignment of raw reads from TCR amplicons, generated using the amplification strategy described in this study, directly to the genome. In summary, a total of 282 TCR gene segments was identified (**Figures 1**–**3**; **Supplementary Table 1**). *VJ-gene-finder* recognized 164 of 169 total functional (F) and open reading frame (ORF) V genes and 67 of 74 total J genes. All V(D)J genes, except TRBV2-4, were located on the reverse strand and each locus comprised only a single C gene. TCR V genes exhibiting ≥75% sequence identity at the nucleotide level were grouped into V families, facilitating classification and analysis.

The TCR α/δ locus spanned ~970 kb on chromosome 27, with TCR δ occupying approximately 310 kb (**Figure 1**). 72 Vα segments were clustered in three families, TRAV1 (T cell receptor alpha Variable family 1) with 48 genes (of which 12 were pseudogenes), TRAV2 with 23 genes and a single TRAV3 gene adjacent to the TCR δ locus (**Figure 1**; **Supplementary Table 1**; **Supplementary Files 1, 2**). All TRAV1 family members except TRAV1-21 (TRAV1 family member number 21), TRAV1-47 and TRAV1-49 encoded a leader peptide in a single exon with the V gene. The majority of TRAV1 member were classified as ORF genes due to a low mean recombination information content (RIC) score of the RSSs (a score that is used to predict physiological RSSs; the score was calculated by *RSSsite* and is defined as the natural logarithm of marginal and joint probability functions of mutually correlated positions), or as pseudogenes containing stop codon or frameshift mutations (39, 68). The α locus contained 64 TRAJ genes, with 7 classified as ORF lacking the conventional “W/FG.G” amino acid motif, or due to a low RIC score of the RSS.

The TCR δ locus was nested between TRAJ and TRAV genes and contained 5 V families, 2 TRDD genes, 2 TRDJ genes and a single TRDC gene. The TRDV1 family, consisting of 41 genes (including 3 pseudogenes), was the largest, while the TRDV2, TRDV3, TRDV4, and TRDV5 families comprised 3, 1, 9 and 3 members, respectively. All TRDV4 genes were ORFs lacking predicted L-PART1 sequences in proximity to the 5’ end. The non-conventional TCR δ-like locus on chromosome 10 comprised a set of single VDJC genes with an IgH V-like VHδ gene, classified ORF due to a low RIC score RSS and the lack of a corresponding L-PART1 sequence that, when spliced to the TRDVH1 gene, would lead to a functional signal peptide.

The TCR β locus, located on chromosome 1 spanning approximately 211 kb, exhibited fewer genes with a total of 16 TRBV genes, that were all functional (**Figure 2**; **Supplementary Table 1**; **Supplementary Files 3, 4**). Those occurred in three families, ordered from 5’ to 3’, with 11 TRBV1 genes followed by 3 TRBV2 genes and one TRBV3 gene, followed by a single D gene, 4 J genes and a single C gene. Additionally, downstream of the C gene, there was a single V gene (TRBV2-4) in an inverted orientation on the forward strand.

In comparison to the TCR β locus, the γ locus on chromosome 2 was more densely packed, containing 53 V genes in four families spread across ~109 kb (**Figure 3**; **Supplementary Table 1**; **Supplementary Files 5, 6**). Notably, a higher proportion of pseudogenes (28% of Vγ genes) was identified in the γ locus compared to the other TCR loci. Nine out of 15 pseudogenes were incomplete fragments of V genes with short stretches of sequence similarity and a predicted RSS, leading to a low-confidence assignment to a specific V gene family. Most members of the TRGV1 family encoded a single-exon leader peptide, mirroring the structure seen in TRAV1 genes. However, their likelihood of containing functional signal peptides was low, as predicted by *SignalP 6.0*. Consequently, they were classified as ORFs, unless additional defects were detected, prompting classification as pseudogenes. In sum, we detected 8 TRGV1 genes (with 2 pseudogenes), 27 TRGV2 genes (with 8 pseudogenes), 10 TRGV3 genes (no pseudogenes), 8 TRGV4 genes (with 5 pseudogenes), 3 TRGJ genes and 1 TRGC gene.

### 3.2 Evolutionary relationships

**Figure 4:**
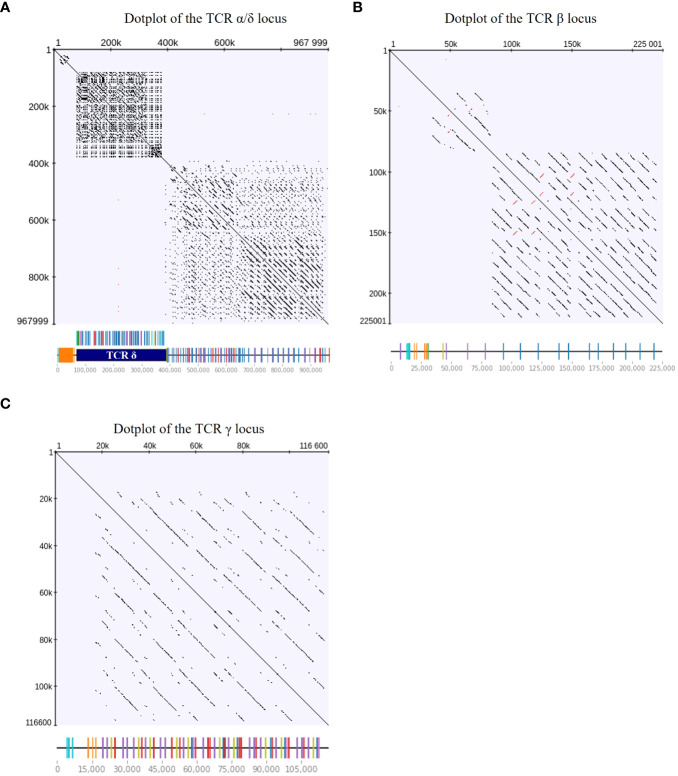
Dotplots of TCR loci indicating long sequence repeats spanning exons and non-coding regions. The DNA sequence of each TCR locus was aligned against itself, indicating sequence repeats with dots and inverted repeats with red dots (minimum repeat length of 100 bp; repeat identity threshold of 100%). Linear locus representations with TCR gene annotations were included on the x axis for reference. Dotplots of the **(A)** TCR α/δ locus, **(B)** TCR β locus, and **(C)** TCR γ locus.

Next, we wanted to analyze the genomic landscape of the TCR loci. Dotplot analysis revealed long stretches of sequence repeats separated by insertions and deletions in all loci, a pattern that is consistent with an evolutionary history shaped by duplication of homology units (**Figure 4**) (69). The regions of sequence similarity were much longer than the VDJ gene segments themselves and spanned across exons and non-coding sequences. We also detected inverted repeats in the TCR β locus. Overall, the TCR loci were low-complexity regions, with redundancy predominantly in genomic regions that contained V genes. Remarkably, repeats in the α/δ locus were contained within regions of the same chain (**Figure 4A**). This separation indicated that sequence duplication events were constrained and occurred separately for each chain, or that inter-chain duplication events in this locus were more ancient.

The overall landscape of repeats in the dotplot (**Figure 4**) reflected the topology of repeating units that could be observed in the genome locus representations (**Figures 1**–**3**). Across the α chain locus, pairs of TRAV1 and TRAV2 genes ~2 kb apart occurred repeatedly, with some variations in the pattern (**Figure 1**). In the dotplot, three clusters of α chain sequence repeats became apparent, a small cluster of repeats around the J genes, a larger cluster of repeats at ~400 - 650 kb with more V genes dispersed between TRAV1/TRAV2 pairs, and, although with some sequence overlap, a distinct cluster with more loosely arranged V genes (**Figure 4A**). The sequence repeats around the Vδ genes formed two clusters: A small cluster at ~310 - 380 kb, predominantly comprised of TRDV1 genes with three TRDV2 and one TRDV1 gene interspersed, and a larger cluster that also contained TRDV4 and TRDV5 family genes (**Figures 1**, **4A**). The repeats around the Vβ genes were separated in two clusters by family, indicating separate evolution of TRBV genes by duplication of homology units and/or early separation from a common ancestral sequence (**Figure 4B**). In the TCR γ locus, long stretches of homology units occurred up to 5 times, and a pattern of repeating units of TRGV4 - TRGV1 - TRGV3 - TRGV2 (1 to 5 TRGV2 genes) with some variations was observed, in agreement with previous reports (**Figures 3**, **4C**) (22, 23).

**Figure 5:**
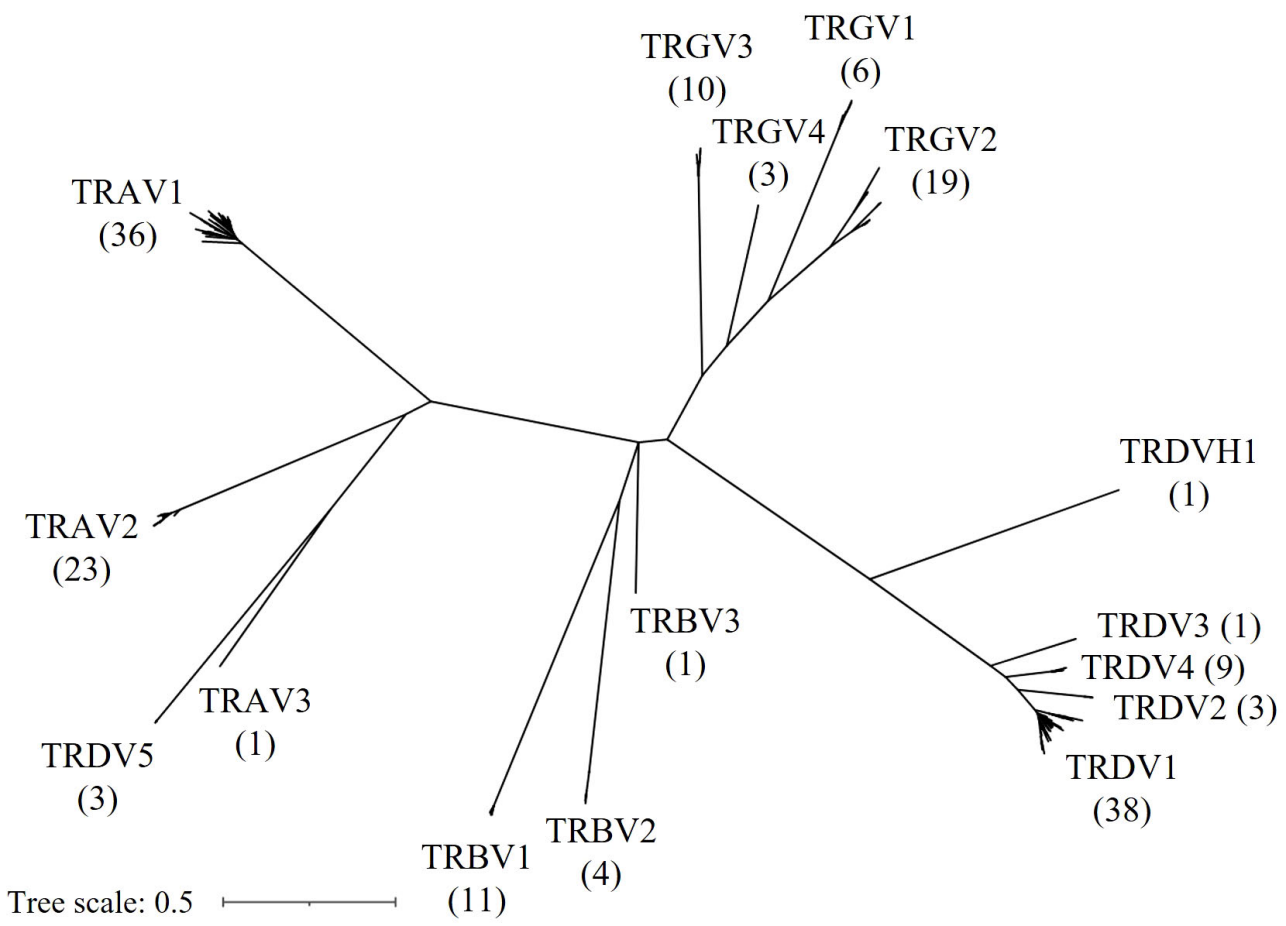
Phylogenetic tree of chicken TRV genes. An unrooted Maximum Likelihood Tree displaying F and ORF TRV genes. Gene names at branch tips were replaced with their respective gene family names and counts in parentheses for improved readability.

Next, we constructed a Maximum Likelihood tree of all F and ORF V genes to characterize the evolutionary relationship between V genes of different TCR loci (**Figure 5**). Reliability of the tree was estimated with the bootstrap method (500 bootstrap replicates). Notably, V sequences separated according to the chain type, except for all three TRDV5 sequences that clustered with the TRA genes and formed a distinct clade with the single TRAV3 gene. In addition, the single TRDVH1 gene from chromosome 10 was quite distinct from TRDV1-4 genes. Overall, the tree supported V family classification, as V families were separated in distinct clades. Members of any given V family shared a high degree of sequence similarity, leading to short branch lengths and poor tree resolution. To better highlight the topology of the tree at the branch tips, unscaled representations of the phylogenetic tree were constructed, with specific nodes collapsed (**Supplementary Figure 3**).

### 3.3 The splenic TCR repertoire

With a complete genome annotation of VDJC gene segments at hand, our goal was to establish an approach for amplification and annotation of the expressed TCR repertoire in chickens. We based our method on a protocol described by Mamedov et al. for 5’RACE with C gene-specific reverse primers and subsequent amplification of TCR sequences by two rounds of PCR (31, 54). New chicken-specific reverse primer sets were established, and a UMI was included in the template switch oligo (TSO = *“SmartNNNNa”*) (**Table 1**). This molecular barcoding of the cDNA enabled deconvolution of PCR copies and duplicates of expressed cDNA during subsequent bioinformatic analysis, leading to more precise quantification.

Next, we wanted to characterize the TCR repertoire in the chicken spleen, as a proof of concept, and to collect baseline data on the TCR diversity in this major lymphoid organ. We amplified TCR sequences from splenic total RNA of three chickens and sequenced purified amplicons with a read length of 2x300 bp. Raw sequences were then analyzed with *MiXCR* (57). As a reference for automated alignment and clonotype assembly of TCR sequences by *MiXCR*, a custom chicken V(D)J library was created from all F and ORF genes annotated in this study. Anchor points that delineate CDR1-CDR3 and surrounding FR were specified for each gene. The CDR3 was defined as the target region in *MiXCR* due to its high sequence variability and its critical role in peptide binding. After processing in *MiXCR*, high-level downstream analysis of 4,000 - 10,000 clonotypes (after downsampling) per chain was performed in *Python* (**Supplementary Figures 4A–D**). The repertoires of each chain were predominantly comprised of unique nucleotide clonotypes that were only represented by a single cDNA molecule in the analyzed pool (only a single UMI barcode per clonotype that passed the reads per UMI thresholds in MiXCR), with some clonotypes exhibiting higher UMI per clonotype counts (**Supplementary Figures 4E–H**). For each chain, we analyzed V(D)J gene and – family usage, CDR3 spectratypes, clonotype rank abundance, publicity, top clonotype sharing and CDR3 amino acid usage.

**Figure 6:**
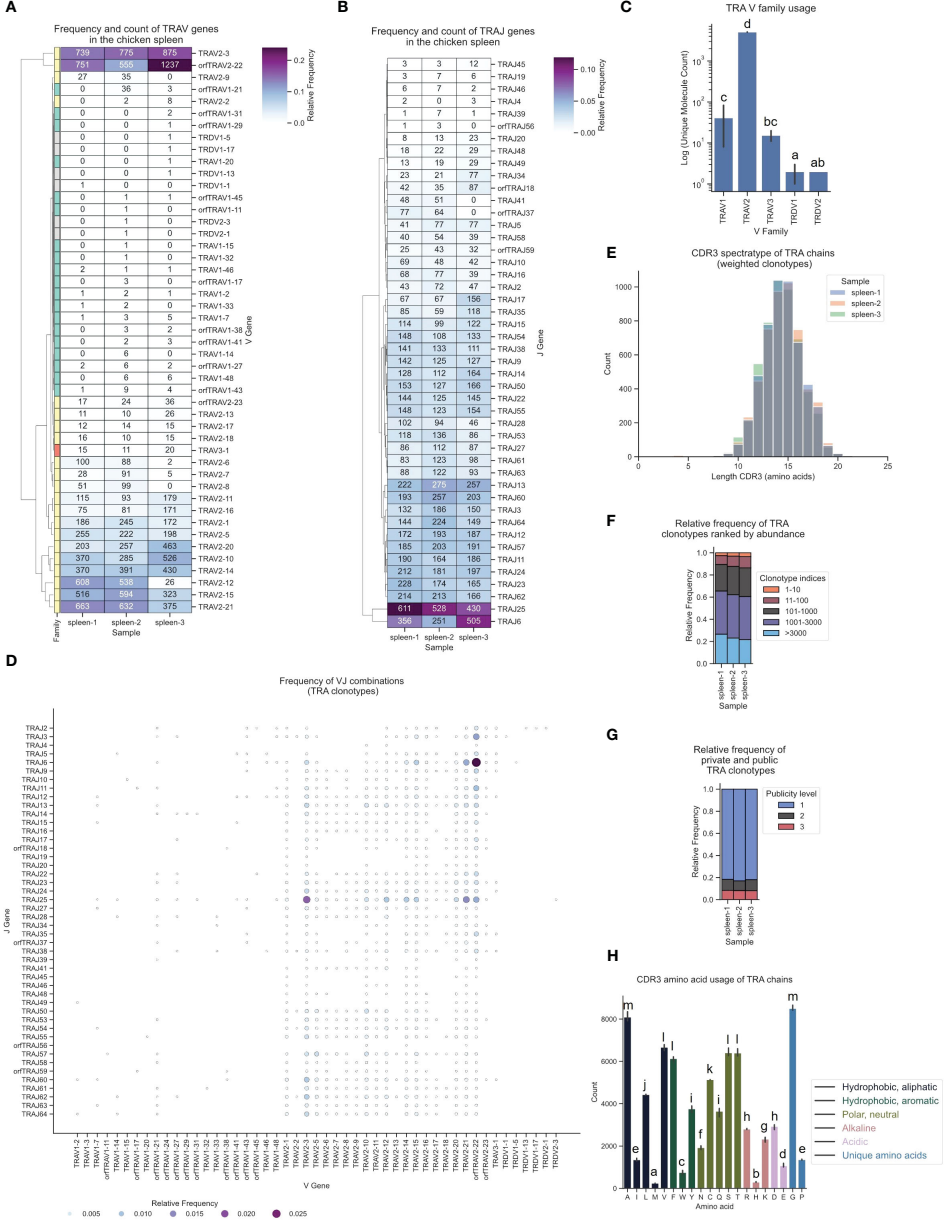
The TCR α chain repertoire expressed in the chicken spleen. TCR α chain amplicons were sequenced at 2x300 bp, followed by alignment and clonotype assembly using *MiXCR* software with a VDJ germline library comprised of the sequences annotated in this study. **(A)** Clustermap displaying V gene utilization for each sample featuring gene counts and color-coded relative frequencies in the heatmap. Row colors denote V family: green (TRAV1), yellow (TRAV2), red (TRAV3), grey (non-TRA V gene). **(B)** Clustermap displaying J gene utilization for each sample featuring gene counts and color-coded relative frequencies in the heatmap. **(C)** Log count of TCR clonotypes grouped by V family in the TCR α chain repertoire. **(D)** Bubble grid plot illustrating relative frequencies of V-J pairings, indicated by bubble size and color-coding. **(E)** CDR3 spectratype displaying the distribution of CDR3 amino acids lengths with frequency-weighted counts. **(F)** Rank abundance plot depicting the proportion of the TCR repertoire occupied by clonotype groups arranged by clonotype abundance rank. **(G)** Proportional abundance of clonotypes categorized by their prevalence across three samples. **(H)** Barplot showing mean counts of individual amino acids in the CDR3. **(C, H)** Mean ± 95% confidence intervals; Means not sharing any letter are significantly different by the Tukey-test at the 5% level of significance.

Strikingly, despite a lower count of TRAV2 genes in the genome (23 F and ORF genes vs. 36 TRAV1), αβ T cells in the spleen predominantly expressed TRAV2 genes (> 90%), while TRAV1 genes and the single TRAV3 gene were detected at very low frequency (**Figures 6A, C**; **Supplementary Figure 5A**). This contradicted a simple positive correlation between gene count and gene expression, indicating that other regulatory mechanisms likely contributed to this strong V gene expression bias. One such regulatory factor could be the RSS sequence, since a high proportion of TRAV1 genes were ORFs with a low RIC score RSS (**Supplementary Table 1**). Individual TRDV1 and TRDV2 genes formed V α/δ chimeric receptors with TCR α genes, as was previously reported by Liu et al., with chimeric receptors detected at very low frequency in all three samples (**Figures 6A, C**). J gene utilization also strongly favored specific J genes, of which TRAJ25 and TRAJ6 had the highest number of transcripts (**Figure 6B**; **Supplementary Figure 6A**). The combinations of VJ segments with the highest expression were TRAV2-22 - TRAJ6 and TRAV2-3 - TRAJ25 (**Figure 6D**; **Supplementary Figure 7**). The TCR α CDR3 spectratype was calculated and showed a Gaussian-like distribution (centered at 14 - 15 amino acids) indicative of an unbiased TCR repertoire without dominant clonally expanded clonotypes (18, 70, 71) (**Figure 6E**). The phenotype of an unbiased repertoire was reinforced by the distribution of clonotype index groups based on their relative frequencies, wherein all clonotypes within the TCR repertoire were ranked by abundance and grouped according to their indices (**Figure 6F**). The analysis revealed that the highest-ranked clonotypes accounted for a minor fraction of the overall repertoire. Furthermore, the color-coded TCR spectratypes representing the proportions of the top 10 most prevalent V genes (**Supplementary Figures 8A–C**) exhibited distributions typical of unbiased repertoires. Roughly 10% of the TCR α repertoire was occupied by “public” clonotypes (present in all three samples), ~5-10% by clonotypes expressed in 2 samples and >80% were “private” TCR clonotypes (**Figure 6G**). Interestingly, the majority of the top 10 clonotypes in each sample were also present at similar frequencies in the two other samples (**Supplementary Figures 9A–C**). None of the clonotypes exhibited expansion beyond 0.5% of the repertoire. Converging CDR3s, representing distinct nucleic acid sequences encoding identical CDR3 amino acid sequences, constituted approximately 20% of the TCR α repertoire (**Supplementary Figures 10A, B**). A maximum of 12 distinct sequences encoding the same CDR3 were identified, serving as a potential biomarker for antigen-specific T cell responses (72). The amino acid composition of the CDR3 showed that Glycine and Alanine were most prevalent, with an overall high proportion of polar neutral amino acids, and low frequencies of acidic and alkaline amino acids (**Figure 6H**).

**Figure 7:**
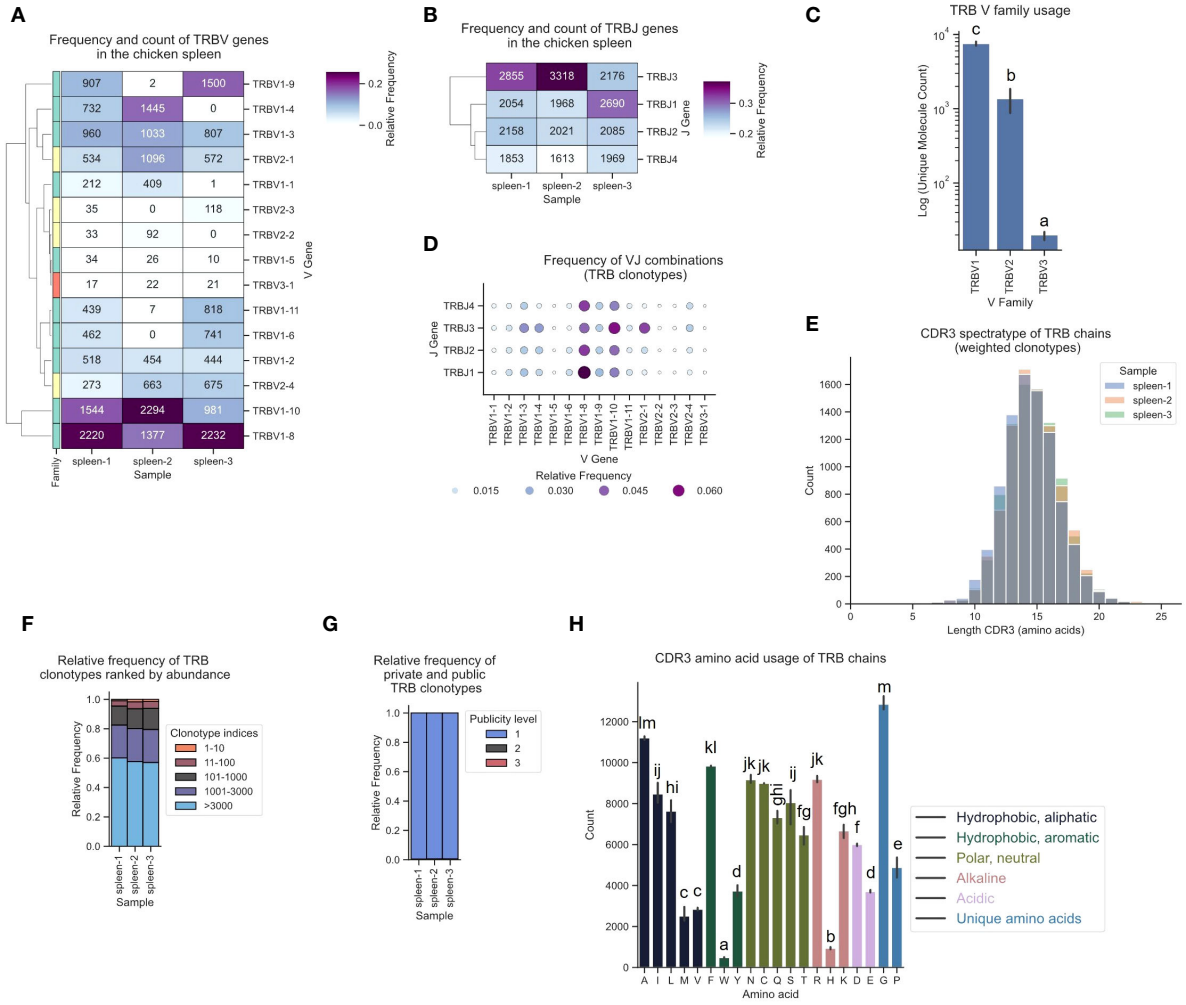
The TCR β chain repertoire expressed in the chicken spleen. TCR β chain amplicons were sequenced at 2x300 bp, followed by alignment and clonotype assembly using *MiXCR* software with a VDJ germline library comprised of the sequences annotated in this study. **(A)** Clustermap displaying V gene utilization for each sample featuring gene counts and color-coded relative frequencies in the heatmap. Row colors denote V family: green (TRBV1), yellow (TRBV2), red (TRBV3). **(B)** Clustermap displaying J gene utilization for each sample featuring gene counts and color-coded relative frequencies in the heatmap. **(C)** Log count of TCR clonotypes grouped by V family in the TCR β chain repertoire. **(D)** Bubble grid plot illustrating relative frequencies of V-J pairings, indicated by bubble size and color-coding. **(E)** CDR3 spectratype displaying the distribution of CDR3 amino acids lengths with frequency-weighted counts. **(F)** Rank abundance plot depicting the proportion of the TCR repertoire occupied by clonotype groups that were categorized based on clonotype abundance rank. **(G)** Proportional abundance of clonotypes categorized by their prevalence across three samples. **(H)** Barplot showing mean counts of individual amino acids in the CDR3. **(C, H)** Mean ± 95% confidence intervals; Means not sharing any letter are significantly different by the Tukey-test at the 5% level of significance.

The corresponding TCR β repertoire primarily consisted of around 80-90% TRBV1-family clonotypes, approximately 10-20% sequences derived from TRBV2, and a minority of TCRs featuring the TRBV3-1 gene (**Figure 7C**). In the observed T cell repertoire, the most frequently utilized V and J genes were TRBV1-8, TRBV1-10, TRBJ3 and TRBJ1, with high expression levels across all J genes (**Figures 7A, B**; **Supplementary Figures 5B, 6B**). TRBV1-8 was predominantly paired with TRBJ1 (**Figure 7D**; **Supplementary Figure 11**). The repertoire was unbiased with no preferentially expanded clones (≥0.5% as defined by Dascalu et al.) and a low degree of convergence (**Figures 7E, F**; **Supplementary Figures 8D–F, 9D–F, 10C–D**) (29). The ranked clonotype abundance distribution closely mirrored the clonal homeostasis proportions reported by Dascalu et al. for the spleen, indicative of a large proportion of naïve T cells (**Figure 7F**). Minimal overlap was observed among the top 10 most prevalent clonotypes when compared across the other two samples and virtually all clonotypes were private (**Figure 7G**; **Supplementary Figures 9D–F**). The amino acid distribution in the CDR3 was overall comparable to α chain CDR3s, with some variation, including a higher proportion of Isoleucine (I), Asparagine (N) and Arginine (R), and relatively fewer Valine (V), Serine (S) and Threonine (T) residues (**Figure 7H**).

**Figure 8:**
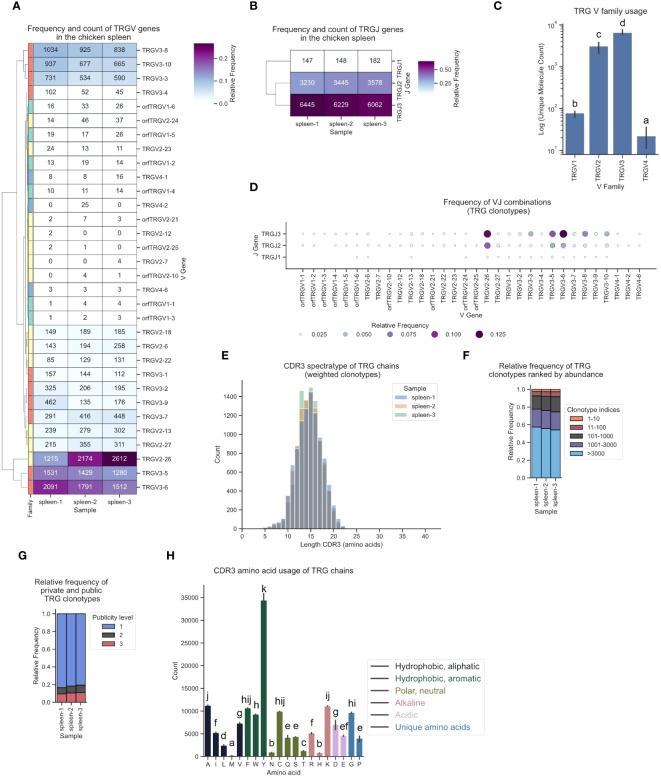
The TCR γ chain repertoire expressed in the chicken spleen. TCR γ chain amplicons were sequenced at 2x300 bp, followed by alignment and clonotype assembly using *MiXCR* software with a VDJ germline library comprised of the sequences annotated in this study. **(A)** Clustermap displaying V gene utilization for each sample featuring gene counts and color-coded relative frequencies in the heatmap. Row colors denote V family: green (TRGV1), yellow (TRGV2), red (TRGV3), blue (TRGV4). **(B)** Clustermap displaying J gene utilization for each sample featuring gene counts and color-coded relative frequencies in the heatmap. **(C)** Log count of TCR clonotypes grouped by V family in the TCR γ chain repertoire. **(D)** Bubble grid plot illustrating relative frequencies of V-J pairings, indicated by bubble size and color-coding. **(E)** CDR3 spectratype displaying the distribution of CDR3 amino acids lengths with frequency-weighted counts. **(F)** Rank abundance plot depicting the proportion of the TCR repertoire occupied by clonotype groups arranged by clonotype abundance rank. **(G)** Proportional abundance of clonotypes categorized by their prevalence across three samples. **(H)** Barplot showing mean counts of individual amino acids in the CDR3. **(C, H)** Mean ± 95% confidence intervals; Means not sharing any letter are significantly different by the Tukey-test at the 5% level of significance.

The TCR γ repertoire displayed a distinct hierarchical pattern in gene utilization, featuring infrequent TCRs from TRGV1 and TRGV4, moderate levels of TRGV2-derived sequences, and a high frequency (60 - 80%) of clonotypes originating from TRGV3 family V genes (**Figures 8A, C**). Similarly, TRGJ1, TRGJ2 and TRGJ3 exhibited analogous trends, with mean frequencies of 1.6%, 34.8% and 63.6%, respectively (**Figure 8B**; **Supplementary Figure 6C**). The most frequently expressed Vγ genes were TRGV2-26, TRGV3-6, and TRGV3-5, each predominantly paired with TRGJ3 (**Figures 8A, D**; **Supplementary Figures 5C, 12**). The CDR3 spectratype resembled an unbiased repertoire with a Gaussian-like distribution, featuring a long tail of low-frequency “ultralong” CDR3γs, as previously described by Zhang et al. (**Figures 8E**; **Supplementary Figures 8G–I**) (23). The top 10 clonotypes collectively represented approximately 2 - 2.5% of the TCRs. Individual clonotypes were moderately expanded, occupying up to 0.63% of the repertoire space. The relative frequency of the most frequent clonotypes was strikingly similar across all three samples (**Figure 8F**; **Supplementary Figures 9G–I**). The distribution of public and private clonotypes mirrored that of the TCR α sequences, comprising 10% found across three samples, while ~80% were private TCRs (**Figure 8G**). A substantial level of convergence was evident, with up to 13 distinct clonotypes encoding identical CDR3 sequences, accompanied by a notable publicness among convergent clonotypes (**Supplementary Figures 10E, F**). The CDR3 amino acid utilization was strongly biased towards tyrosine residues, representing 23.9% of all amino acids in γ chains TCRs (**Figure 8H**).

**Figure 9:**
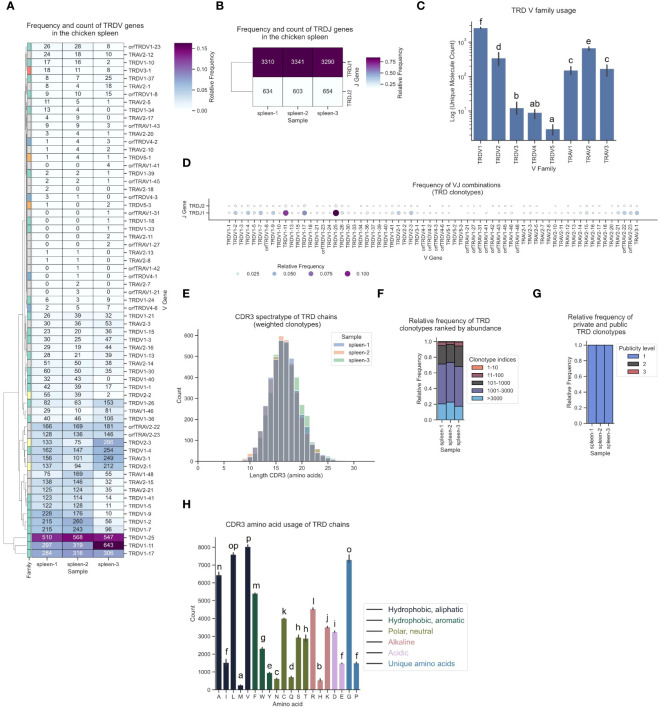
The TCR δ chain repertoire expressed in the chicken spleen. TCR δ chain amplicons were sequenced at 2x300 bp, followed by alignment and clonotype assembly using *MiXCR* software with a VDJ germline library comprised of the sequences annotated in this study. **(A)** Clustermap displaying V gene utilization for each sample featuring gene counts and color-coded relative frequencies in the heatmap. Row colors denote V family: green (TRDV1), yellow (TRDV2), red (TRDV3), blue (TRDV4), orange (TRDV5), grey (non-TRD V gene). **(B)** Clustermap displaying J gene utilization for each sample featuring gene counts and color-coded relative frequencies in the heatmap. **(C)** Log count of TCR clonotypes grouped by V family in the TCR δ chain repertoire. **(D)** Bubble grid plot illustrating relative frequencies of V-J pairings, indicated by bubble size and color-coding. **(E)** CDR3 spectratype displaying the distribution of CDR3 amino acids lengths with frequency-weighted counts. **(F)** Rank abundance plot depicting the proportion of the TCR repertoire occupied by clonotype groups arranged by clonotype abundance rank. **(G)** Proportional abundance of clonotypes categorized by their prevalence across three samples. **(H)** Barplot showing mean counts of individual amino acids in the CDR3. **(C, H)** Mean ± 95% confidence intervals; Means not sharing any letter are significantly different by the Tukey-test at the 5% level of significance.

Various V gene families contributed to TCR δ sequences, with high expression of TRDV1-derived sequences, intermediate levels of TRDV2-family TCRs and infrequent expression of TRDV3, TRDV4 and TRDV5 (**Figures 9A, C**). Notably, approximately every 4^th^ - 5^th^ TCR was a chimeric receptor formed by somatic DNA recombination of TCR δ DJ-C genes with TRAV1, TRAV2 or TRAV3 V genes. In this dataset, 28 Vα genes contributed to the TCR δ repertoire (**Figure 9D**). Top V genes TRDV1-25 and TRDV1-11 were predominantly recombined to TRDJ1 (**Figures 9A, B, D**; **Supplementary Figures 5D, 6D, 13**). Similar to the other chains, the TCR δ repertoire was phenotypically unbiased with no dominant clonotypes (**Figures 9E, F**; **Supplementary Figures 8J–L, 9J–L**). The CDR3 regions were longer in δ chain TCRs (distribution centered at 16-17 amino acids) and most clonotypes were private with low convergence levels (**Figures 9E, G**; **Supplementary Figures 10G, H**). Unlike in the γ chain CDR3, the tyrosine content in TCR δ CDR3s was low, indicating that the two chains each contribute different binding properties to the CDR3 peptide binding groove in many γδ TCRs (**Figure 9H**). Our analysis pipeline consistently delivered reproducible results across three biological replicates for all TCR chains.

In summary, we delineated fundamental traits of the unbiased αβ and γδ T cell repertoires in the chicken spleen. The analysis encompassing the chicken TCR V(D)J germline genes along with the corresponding expressed TCR sequences has, for the first time, been comprehensively established across all four TCR chains. Our findings lay the groundwork for further investigations that will elucidate chicken T cell functions in infection and immunity.

## 4 Discussion

TCR repertoire sequencing, in conjunction with advances in NGS technology, has emerged as a key for understanding T cell biology and the dynamic composition of the T cell population. Most human repertoire studies focused on αβ T cells, because γδ T cells are present at low frequencies in the peripheral blood and other tissues. Specifically, the β chain CDR3 is the main target of human repertoire sequencing projects due to its high combinatorial diversity and important role in peptide binding (73). Considering the high frequency of γδ T cells and the overall poorly characterized T cell responses in chickens, we concluded that an approach encompassing both αβ and γδ T cell repertoires is essential. This comprehensive analysis is crucial for addressing questions regarding the roles of chicken T cells in homeostasis, vaccination, and infection. However, this analysis has been hampered by a lack of publicly available annotations for TCR α/δ V(D)J sequences.

To address this gap of knowledge, we established a comprehensive and standardized annotation across all TCR loci in the high-quality Huxu chicken genome assembly, including the TCR α/δ hybrid locus on chromosome 27 (**Figures 1**–**3**) (34). We initially attempted to annotate the TCR loci in the current reference genomes *bGalGal1.mat.broiler.GRCg7b* and *bGalGal1.pat.whiteleghornlayer.GRCg7w_WZ* (two haplotypes of a cross between a modern broiler breeder mother and White Leghorn father), but noticed that the TCR sequences on chromosome 27 appeared to be incorrectly distributed between the two haplotypes (data not shown). In need of a tool to quickly assess the TCR loci in alternative genomes, we developed *VJ-gene-finder*, enabling automated VJ gene extraction. With an increasing number of new high-quality genomes being released for various chicken breeds, this tool represents a significant step towards an efficient and reproducible annotation of chicken TCR V(D)J genes across genomic datasets (between January and November 2023, 7 new genome assemblies were published on NCBI: https://www.ncbi.nlm.nih.gov/datasets/genome/?taxon=9031).

*VJ-gene-finder* (v0.1) identified 97% of F&ORF V genes and 91% of all F&ORF J genes that were characterized as part of this study. Yet, *VJ-gene-finder* lacks functionality for identification of D genes and pseudogenes. With the chicken genome bearing only three known D genes (1 TRBD and 2 TRDD genes), however, manual annotation is straightforward. Annotation of pseudogenes, on the other hand, is not strictly required for TCR analysis since pseudogenes lack essential features and do not contribute to the expressed repertoire. We manually curated *VJ-gene-finder* results, involving the removal of unspecific hits outside of the TCR loci, refining the start - and end positions of genes and associated features, as well as classification of functionality. Insights gained from the annotation process will guide potential iterations of *VJ-gene-finder*, facilitating its further development and refinement. The current algorithm was specifically designed for chicken TCR sequences. However, the search criteria rely on broadly conserved features across species and can be readily adapted for diverse species. For this purpose, *VJ-gene-finder* is now freely available as open-source software.

This work offers crucial insights into chicken TCR loci. Additionally, we present a comprehensive report on our chicken-specific TCR repertoire pipeline, including the repertoire in the spleen. We incorporated molecular barcoding in our pipeline for more precise quantification of the expressed TCR chains. Collectively, this study will lay the foundation for future TCR repertoire analyses in chickens, facilitating systematic comparisons across tissues, chicken breeds (such as broiler and layer chickens), and exploring repertoire dynamics during infection.

The V(D)JC annotation outlined in this study extends beyond previous annotations of the TCR α/δ locus (14, 19). Genome annotation results can vary based on genome quality and annotation methods. Given the rapid advancements in these domains, this discussion focuses on more recent studies. Parra and Miller annotated the chicken TCR α/δ in the red jungle fowl genome released in 2004 (14). They described 41 Vα1 genes, 19 Vα2 genes, 36 Vδ1 genes, 2 Dδ genes, 2 Jδ genes, and 48 Jα genes within a ~800 kb locus on chromosome 27. Liu et al. sequenced BAC clones covering most of the TCR α/δ locus and annotated VDJC genes, with gaps between BAC sequences filled using the *galGal4* genome. They detected 54 Vα genes (10 families) and 31 Vδ genes (4 families), 2 Dδ genes, 2 Jδ genes and 67 Jα genes in the ~800 kb locus. Unfortunately, both studies lacked a detailed map of gene positions in the reference sequence, and the VDJ sequences themselves were also not published. In comparison, the locus in the Huxu genome was larger, spanning almost 1000 kb, while the overall architecture was similar. Accordingly, the locus contained a higher number of V genes compared to previous reports by Parra and Miller, and Liu et al., with a total of 72 Vα genes in three families and 57 Vδ genes in 5 families, and no major differences in J and D genes (**Figure 1**; **Supplementary Table 1**). Liu et al. described two Vα genes within the δ locus, which likely correspond to TRDV5 family members in our annotation (**Figure 1**). The phylogenetic tree indicated that the sequences were evolutionarily more closely related to Vα genes (**Figure 4**). However, despite the proximity, no expression of these genes was identified within the α repertoire, and the sequences were located within the δ locus, prompting our classification of them as TRDV genes. To facilitate better comparability, comprehensive sequence information is provided in the **Supplementary Data** section.

A recent study by Zhang *at al.* characterized the TCR β locus in the red jungle fowl *GRCg6a* genomes, reporting more V genes than existing annotations (18–20). The genomic map of the TCR β locus in the Huxu genome closely resembled the locus structure reported by Zhang et al., with a total of 11 Vβ1 genes, 4 Vβ2 genes (one positioned downstream of the C gene in an inverted manner), 1 Vβ3 gene, 1 Dβ gene and 4 Jβ genes (**Figure 2**) (20). One Vβ1 gene was a pseudogene in the *GRCg6a* assembly, while all Vβ genes were functional in the Huxu genome. Zhang et al. named genes in ascending order away from the C gene. We propose to adhere to earlier conventions, naming the genes in 5’ - 3’ direction leading up to the C gene in the genomic organization (18, 19, 74). A comparative analysis of TCR β V(D)J sequences reported by Zhang et al. with those annotated in our study revealed complete nucleotide identity only in D and J genes, along with the Vβ3 gene (**Supplementary Table 2**). Notably, direct pairwise comparisons between red jungle fowl genes and Huxu genes did not consistently result in a one-to-one match; some Huxu genes emerged as the best match multiple times, while others were never identified as the best match. This variability suggests variations in TCR genes between chicken lines.

Three recent studies published annotations of the TCR γ locus, with varying numbers of V genes and families (19, 22, 23). Liu et al. reported 37 Vγ genes (11 families) based on BAC sequencing and the *galGal4* genome. The other two studies were both based on the *GRCg6a* genome assembly. Dixon et al. partially addressed a discrepancy concerning a potentially duplicated 15 kb sequence fragment containing 13 V genes in the *GRCg6a* genome assembly, which seemed to have been excluded from the published annotation. This duplication was not discussed in the other study. Nonetheless, both studies reached similar conclusions regarding the number of TCR genes: Zhang et al. identified 44 Vγ in 6 distinct subgroups (*GRCg6a* genome), while Dixon et al. described 40 Vγ genes in 4 families. In this study, we characterized 53 Vγ genes, 9 of which were short remnant fragments with an RSS. Those would have likely been disregarded in other studies, leading to 44 remaining full-length Vγ genes organized in 4 families. These numbers overall align with previous work. In summary, multiple studies have provided consistent findings regarding the general organization of the locus, including the counts of D, J and C genes. However, discrepancies arise notably in the count of V genes. Comprehensive reporting of annotated sequences and the corresponding reference sequence becomes pivotal for facilitating direct comparisons among studies and, consequently, establishing a standardized nomenclature. For future comparative analyses, we conducted a comparison of TCR γ V(D)J genes reported by Zhang et al. with those annotated in our study (**Supplementary Table 3**). Similar to the TRB sequences, only J genes and some V genes exhibited 100% identity, and the sequences did not consistently match one-to-one in the red jungle fowl and Huxu genomes.

The evolutionary history of TCR loci is characterized by expansion, leading to diversification of the TCR repertoire. At the same time, evolutionary constraints lead to conserved sequences, residues and motifs that are essential for TCR genes, somatic DNA recombination, and the TCR structure. Those were the patterns that we aimed to identify with the search algorithm incorporated in *VJ-gene-finder*. The processes that shaped the evolution of TCR sequences likely were a complex combination of duplications, insertions, deletions, and gene conversion (69). The more distantly related genes in different V families and the highly similar sequences within gene families are an example of ancient and recent duplications. Overall, we found evidence for multiple duplications of specific homology units in all TCR loci.

In the TCR repertoire analyses of the spleen several general patterns emerged. First, the repertoires in the spleen were predominantly unbiased with Gaussian-like spectratypes. Second, clonotypes were predominantly rare, with moderately expanded clonotypes (<1% of the repertoire space) found in γ repertoires, and, to a lesser degree in α repertoires. Third, V and J gene utilization was strongly biased in all chains, except for TRBJ genes that were all commonly expressed in β chains. Several factors could contribute to variable expression, including the sequence of the RSS itself. Moreover, other sequence motifs in upstream noncoding regions of TCR genes, such as CRE and R-tract motifs, could control V gene expression (69). Observed biases in various tissues could also be a function of thymic positive and negative selection, differential homing properties and selective expansion upon tissue-specific stimuli. The fourth pattern was that chimeric receptors occurred frequently in the δ repertoire and infrequently in the α repertoire. Fifth, a significant proportion of γ and α clonotypes was public, while β and δ (the D-gene containing chains) were predominantly private (at this sequencing depth). Sixth, the top 10 most frequent clonotypes occurred at similar frequencies in α and γ repertoires, but not in β and δ chains. In addition, rank abundance plots indicated that the top 100 clone groups occupied larger proportions of the γ and α repertoires, pointing towards overall more (moderately) expanded clones. Several expanded clones, notably within the γ repertoire, exhibited significant convergence, suggesting preferential selection of specific CDR3 amino acid sequences.

Finally, amino acids showed variable prevalence in CDR3s of each chain, with a striking bias towards tyrosine residues in γ chains (**Figure 8H**). While tyrosine residues are not generally overrepresented in human and mouse αβ TCR CDR3s, they are significantly enriched in immunoglobulin CDR3s. Specifically in murine CDR-H3s, the proportion of tyrosine residues reached 25%, akin to the relative frequency observed in chicken γ chains (75–77). This bias was attributed to the advantageous physiochemical properties of the tyrosine aromatic side chains, permitting flexible molecular interactions at the antigen binding sites (75, 78). Consequently, a higher tyrosine content in CDR3s would facilitate binding of a wider range of (structurally diverse) ligands. This raises important questions about the nature of γδ TCR ligands, which may or may not be restricted to peptide-MHCI/II complexes. Ultimately, conducting structural analysis of the chicken γδ TCR, akin to the αβ TCR, and identifying its ligands are essential steps towards analysing the antigen recognition of the γδ TCR (10).

Overall, our repertoire analyses broadly align with previous studies, although care is warranted when direct comparisons are drawn due to inherent biases from differences in experimental design, chicken breeds, genome annotations, tissues and methodology used (20, 22, 23, 29).

Several technical aspects need to be considered in the experimental design. We sequenced between 59172 and 131282 paired end reads per sample and chain. The successful alignment rate in *MiXCR* varied between chains (from ~70% to > 95%), with the lowest success in α chain sequences. Based on an initial analysis of unaligned reads, our preliminary hypothesis is that many non-regular TCR sequences containing introns and/or UTRs were amplified along with functional TCRs during 5’ RACE amplification. Since this is a known phenomenon, a dedicated computational tool for the analysis of irregular TCRs was developed, which aligns TCR sequences directly to the genome (79). In our analysis, we excluded non-regular TCRs because the functional relevance of such alternative transcripts is unclear. Furthermore, a notable portion of sequences exhibited stop codons (≤1% in α and β chains and ≤5% in γ and δ chains) or frameshift mutations (≤8% in α and β chains ≤20% in γ and δ chains). We excluded such non-expressed TCRs in the post analysis. Together, these factors should be considered when estimating the desired sequencing depth. Notably, UMI-based PCR error correction is powerful, but a high sequencing coverage is required (80).

Bulk TCR repertoire analysis, as described here, represents a fast, accurate and powerful method for conducting an in-depth characterization of T cell responses. Compared to emerging single-cell technologies, bulk TCR repertoire sequencing can be used to sequence very deeply for the analysis of low-frequency clones, but the paring of αβ and γδ chains can only be inferred. Regardless of single-cell or bulk analysis, of utmost importance will be to connect TCR sequence information to structural analysis and ligand identification. Studies focusing on antigen-specific responses will play a pivotal role in unravelling the roles of both αβ and γδ T cells within the adaptive immune responses of chickens.

## Data availability statement

The original contributions presented in the study are publicly available. This data can be found here: [NCBI - PRJNA1068558].

## Ethics statement

The animal study was approved by Government of Upper Bavaria, identification code: 55.2-1-54-2532.0-60-2015; June, 2019. The study was conducted in accordance with the local legislation and institutional requirements.

## Author contributions

SF: Conceptualization, Data curation, Formal analysis, Investigation, Methodology, Validation, Visualization, Writing – original draft, Writing – review & editing. MF: Software, Writing – review & editing. BK: Funding acquisition, Project administration, Resources, Supervision, Writing – review & editing. TG: Conceptualization, Funding acquisition, Project administration, Resources, Supervision, Writing – review & editing.
