# Supplementary material for: Unraveling the chicken T cell repertoire with enhanced genome annotation
Source: Front Immunol. 2024 Mar 14;15:1359169. doi: 10.3389/fimmu.2024.1359169 (PMC10972964; doi:10.3389/fimmu.2024.1359169)
Supplement: Supplementary file 7 [file DataSheet_7.zip › Supplementary_Material.docx]

Supplementary Material

Unraveling the chicken T cell repertoire with enhanced genome annotation

Simon P. Früh*, Martin A. Früh, Benedikt B. Kaufer, Thomas W. Göbel*

*** Correspondence:** Thomas W. Göbel: [goebel@lmu.de](mailto:goebel@lmu.de); Simon P. Früh: [s.frueh@fu-berlin.de](mailto:s.frueh@fu-berlin.de)

# Supplementary Data

The annotation csv files contain a list of V, D and J features with chromosomal coordinates in the Huxu genome, and notes related to classification (F, ORF or P).

The fasta files contain nucleic acid sequences of VDJC genes, signal peptides, RSS and splice sites with chromosomal coordinates in the Huxu genome.

**Supplementary File 1:** Chr27_features_annotation.csv

**Supplementary File 2:** Chr27_features_bases.fasta

**Supplementary File 3:** Chr1_features_annotation.csv

**Supplementary File 4:** Chr1_features_bases.fasta

**Supplementary File 5:** Chr2_features_annotation.csv

**Supplementary File 6:** Chr2_features_bases.fasta

# Supplementary Figures and Tables

## Supplementary Figures


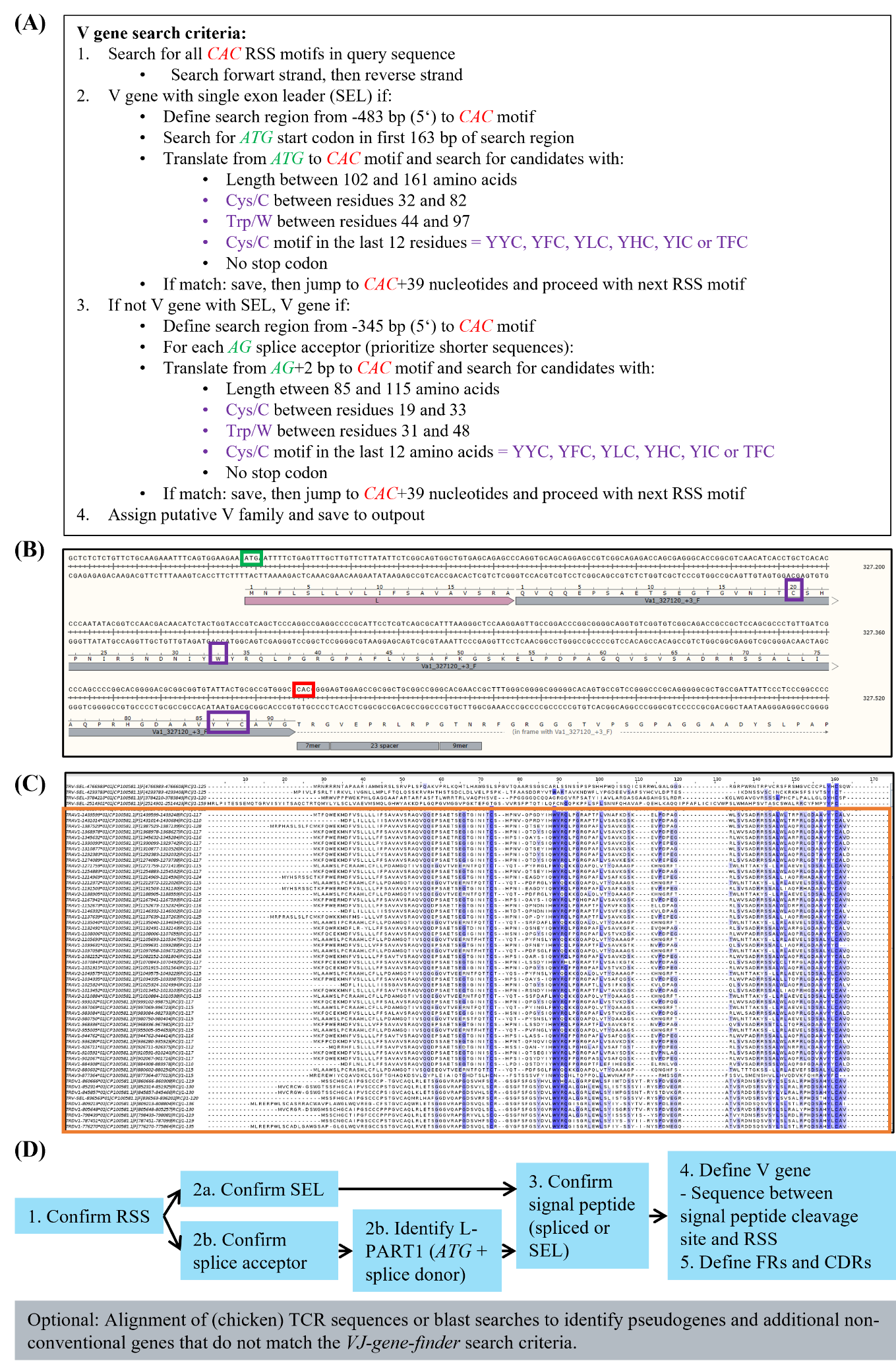


**Supplementary Figure 1.** Annotation pipeline for V genes using *VJ-gene-finder*.

**(A)** General summary of the *VJ-gene-finder* search algorithm for V genes. The search parameters may need to be adjusted for non-chicken query sequences. **(B)** Example of a chicken V gene with color-coded boxes highlighting conserved sequence motifs (colors as in (A)). **(C)** Example of an alignment of *VJ-gene-finder* hits. Actual TCR genes (orange box) can be distinguished from more dissimilar unspecific search hits by alignment, high similarity within TCR genes and the location on the chromosome (location information in sequence name). **(D)** Manual confirmation of (predicted) functionality of recombination signal sites (RSS), splice sites and signal peptides (spliced or from a single exon) is required, and the borders of the V gene may need to be adjusted accordingly. Framework (FR) - and complementarity-determining regions (CDRs) need to be identified manually.


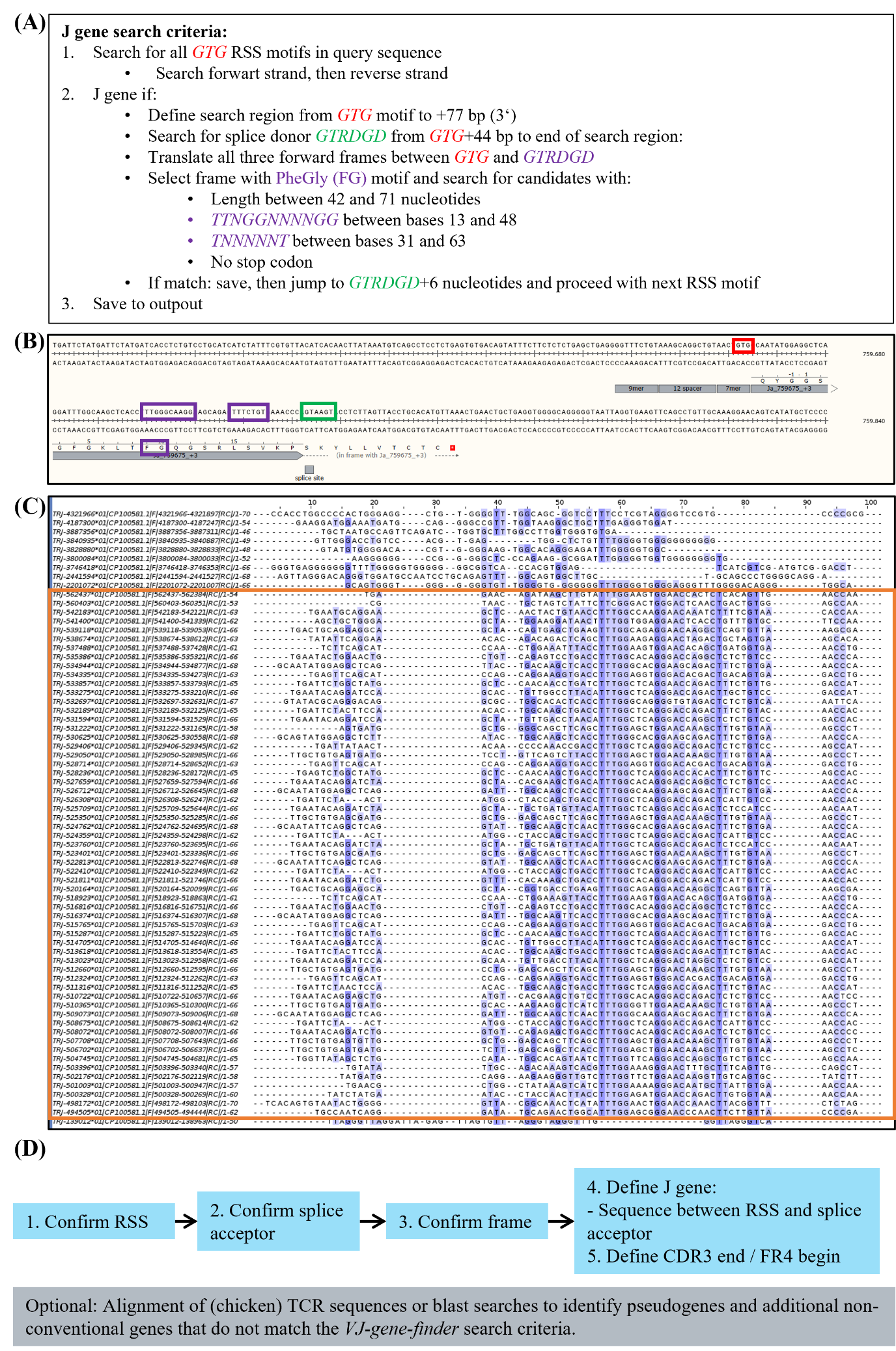


**Supplementary Figure 2.** Annotation pipeline for J genes using *VJ-gene-finder*.

**(A)** General summary of the *VJ-gene-finder* search algorithm for J genes. The search parameters may need to be adjusted for non-chicken query sequences. **(B)** Example of a chicken J gene with color-coded boxes highlighting conserved sequence motifs (colors as in (A)). **(C)** Example of an alignment of *VJ-gene-finder* hits. Actual TCR genes (orange box) can be distinguished from more dissimilar unspecific search hits by alignment, high similarity within TCR genes and the location on the chromosome (location information in sequence name). **(D)** Manual confirmation of (predicted) functionality of recombination signal sites (RSS), splice sites and the open reading frame is required, and the borders of the J gene may need to be adjusted accordingly. The end of complementarity-determining region 3 (CDR3) and the start of framework 4 (FR4) needs to be identified manually.


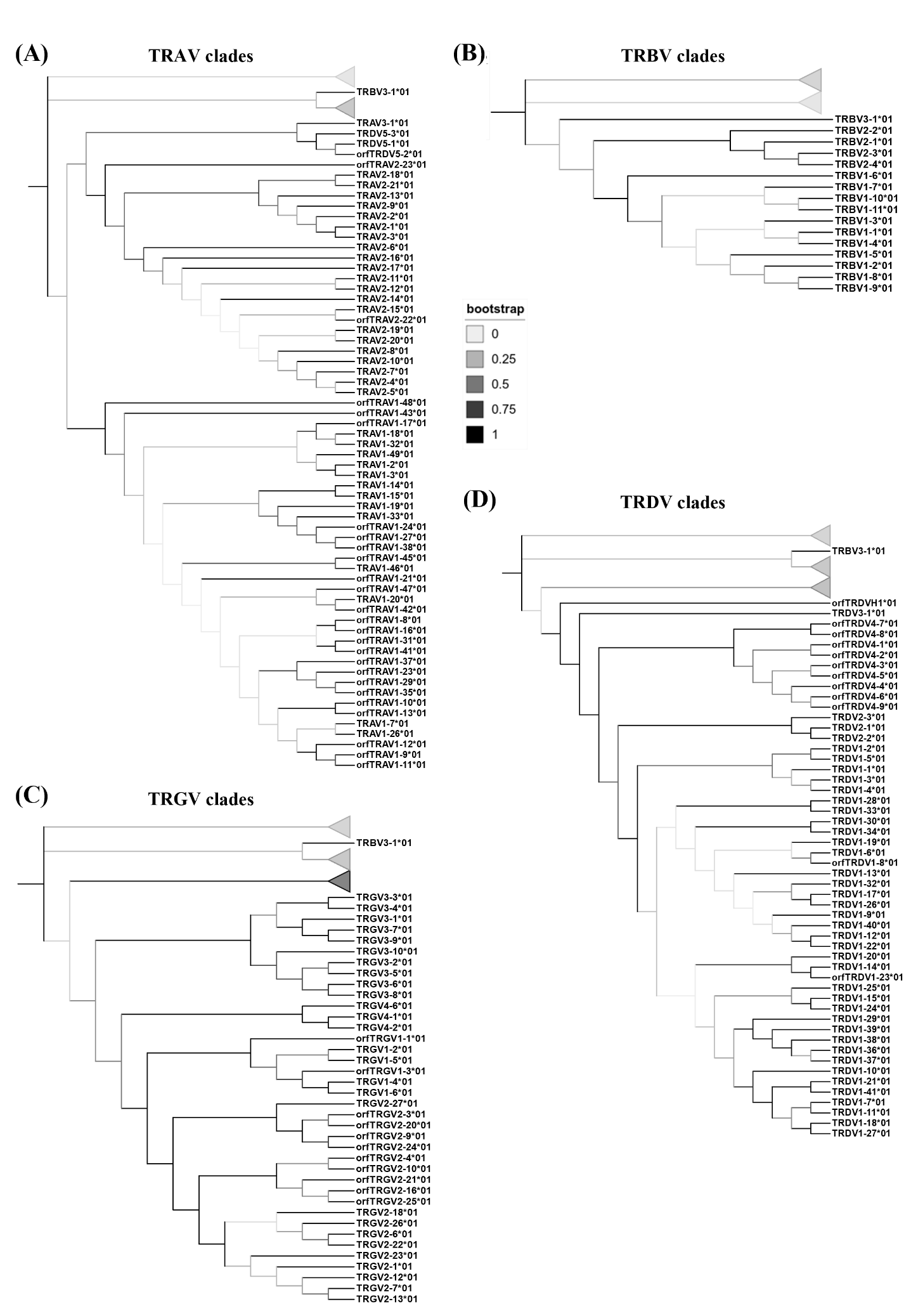


**Supplementary Figure 3.** Unscaled phylogenetic tree of chicken V segments.

Unscaled maximum likelihood tree of F and ORF TRV genes, showing bootstrap support for each branch via greyscale color coding. **(A-D)** Subtree depictions with collapsed nodes for enhanced resolution: **(A)** TRBV, TRGV and TRDV collapsed; **(B)** TRAV, TRGV and TRDV collapsed; **(C)** TRAV, TRBV and TRDV collapsed; and **(D)** TRAV, TRBV and TRGV collapsed.


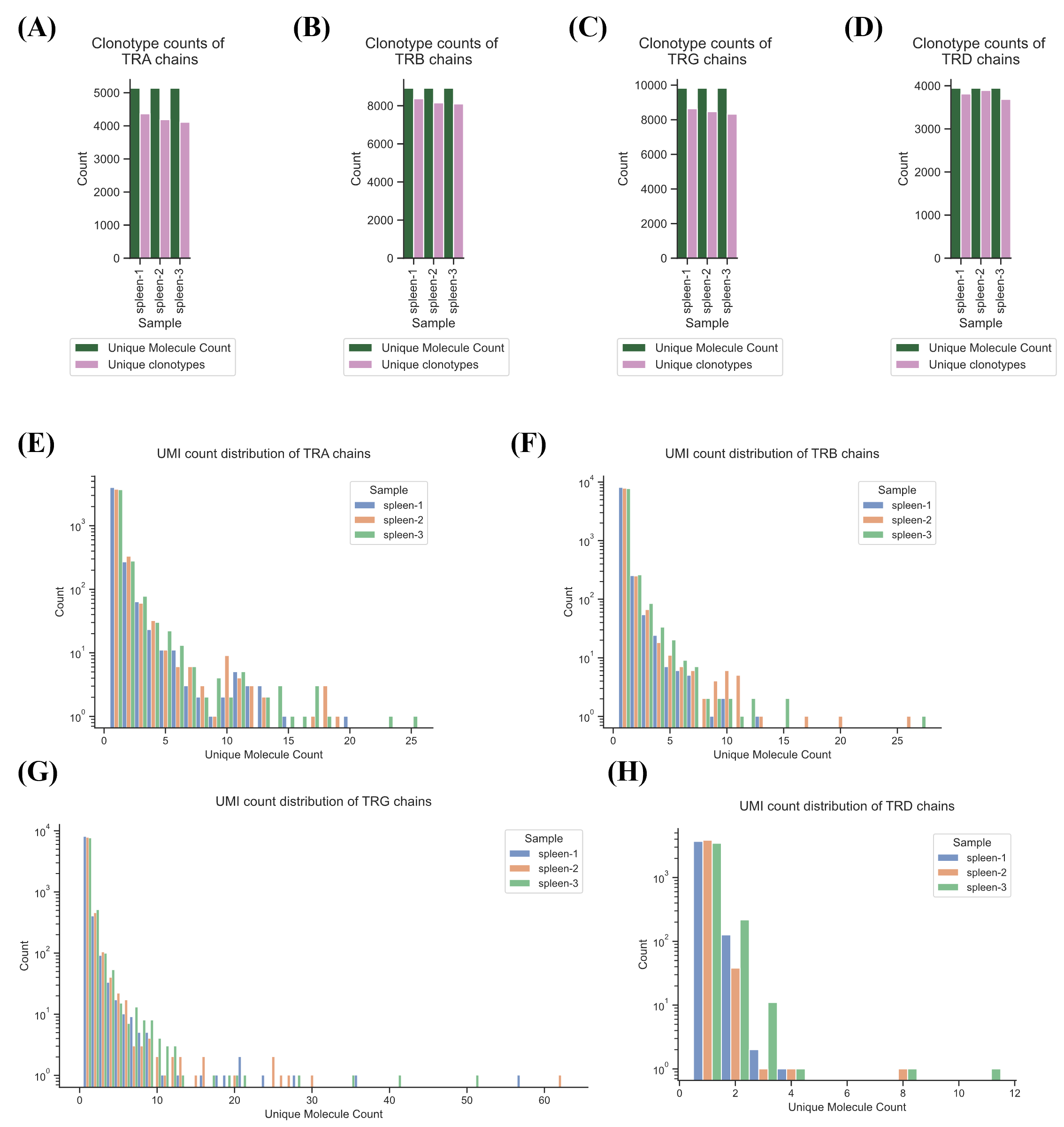


**Supplementary Figure 4.** Clonotype count and UMI statistics of the TCR repertoires analyzed.

Splenic TCR repertoires were amplified and sequenced. **(A-D)** Barplots displaying the total sum of clonotypes weighted by the number of UMIs, and the count of unique clonotypes in **(A)** TCR α, **(B)** TCR β, **(C)** TCR γ and **(D)** TCR δ chain repertoires depicted for each sample. **(E-H)** Histograms showing the distribution of UMI counts per clonotype in **(E)** TCR α, **(F)** TCR β, **(G)** TCR γ and **(H)** TCR δ chain repertoires.

**
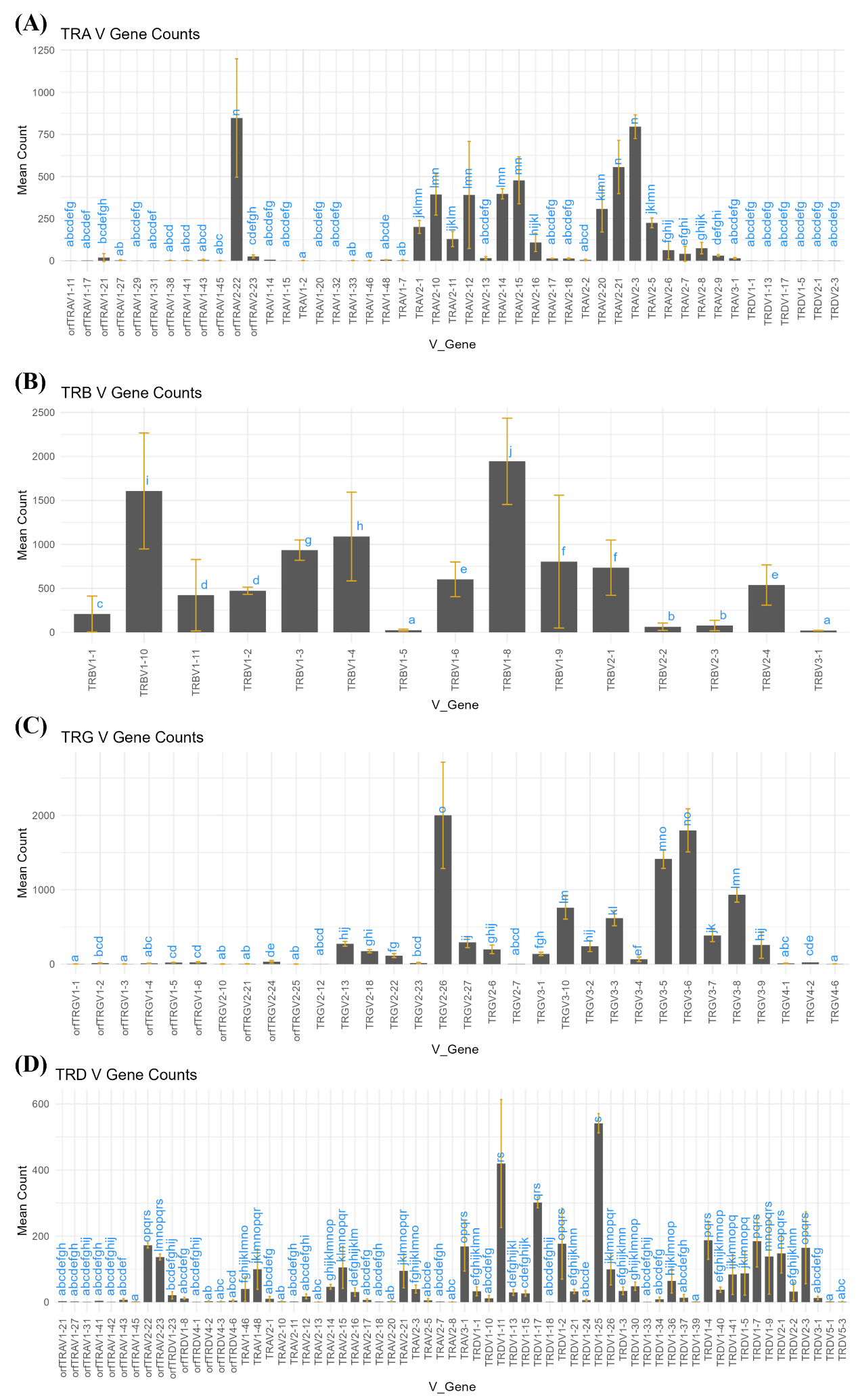
**

**Supplementary Figure 5.** V gene usage in the chicken spleen.

V gene counts of **(A)** TRA, **(B)** TRB, **(C)** TRG and **(D)** TRD clonotypes. Mean ± SD; Means not sharing any letter are significantly different by the Tukey-test at the 5% level of significance.

**
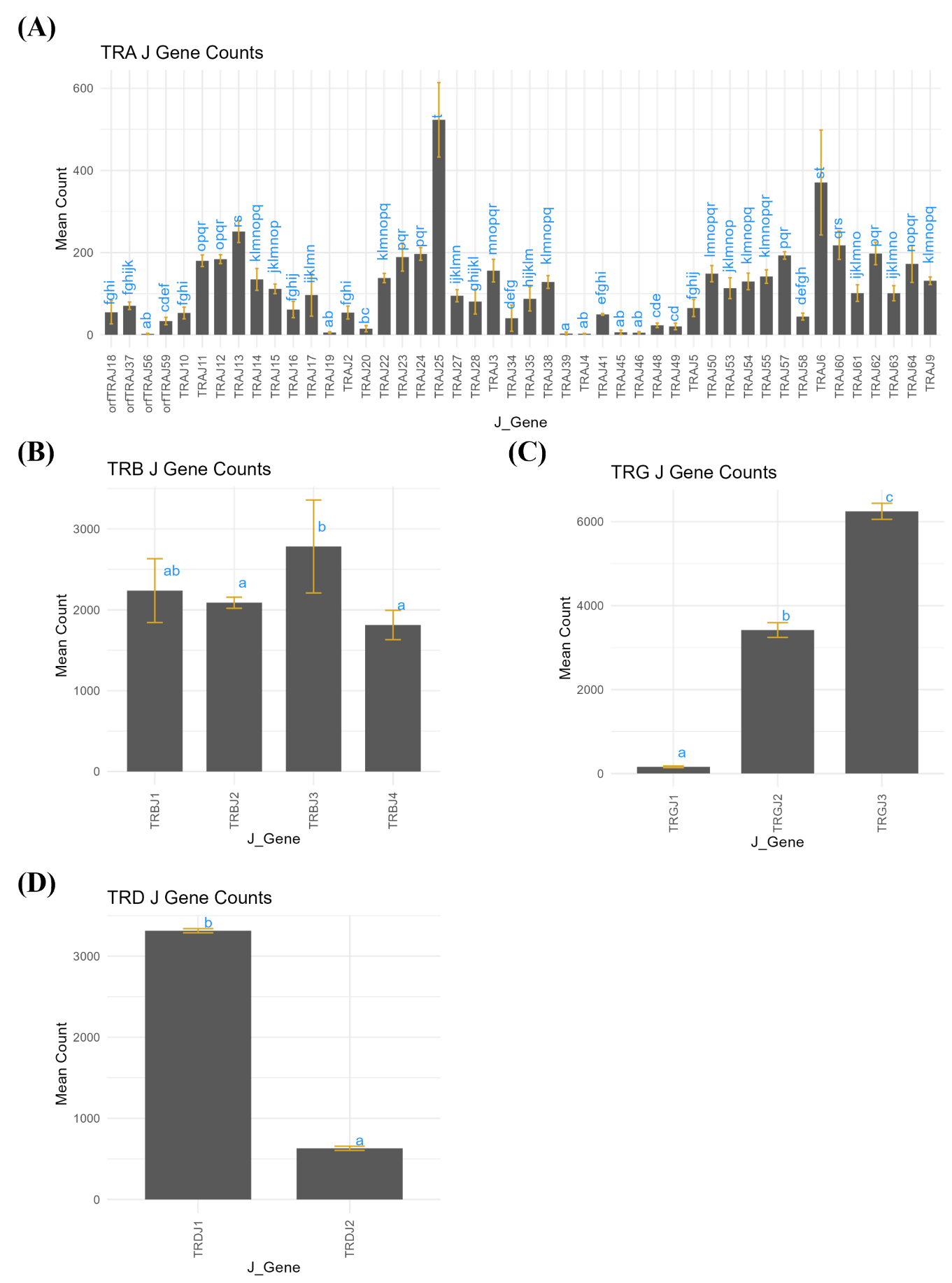
**

**Supplementary Figure 6.** J gene usage in the chicken spleen.

J gene counts of **(A)** TRA, **(B)** TRB, **(C)** TRG and **(D)** TRD clonotypes. Mean ± SD; Means not sharing any letter are significantly different by the Tukey-test at the 5% level of significance.


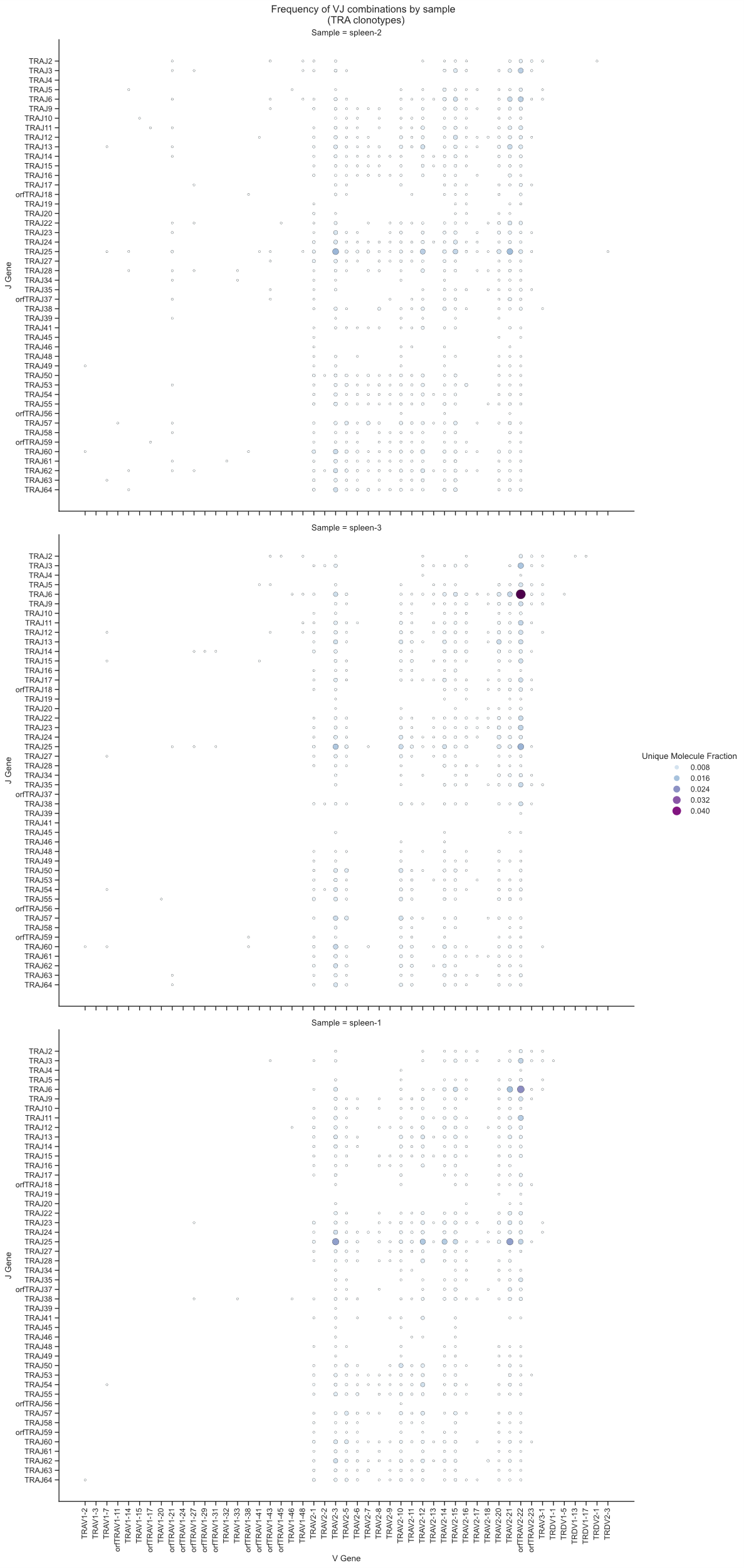


**Supplementary Figure 7.** Combinations of V and J genes in TCR α clonotypes depicted separately for each sample.

Bubble grid plot illustrating relative frequencies of V-J pairings, indicated by bubble size and color-coding.


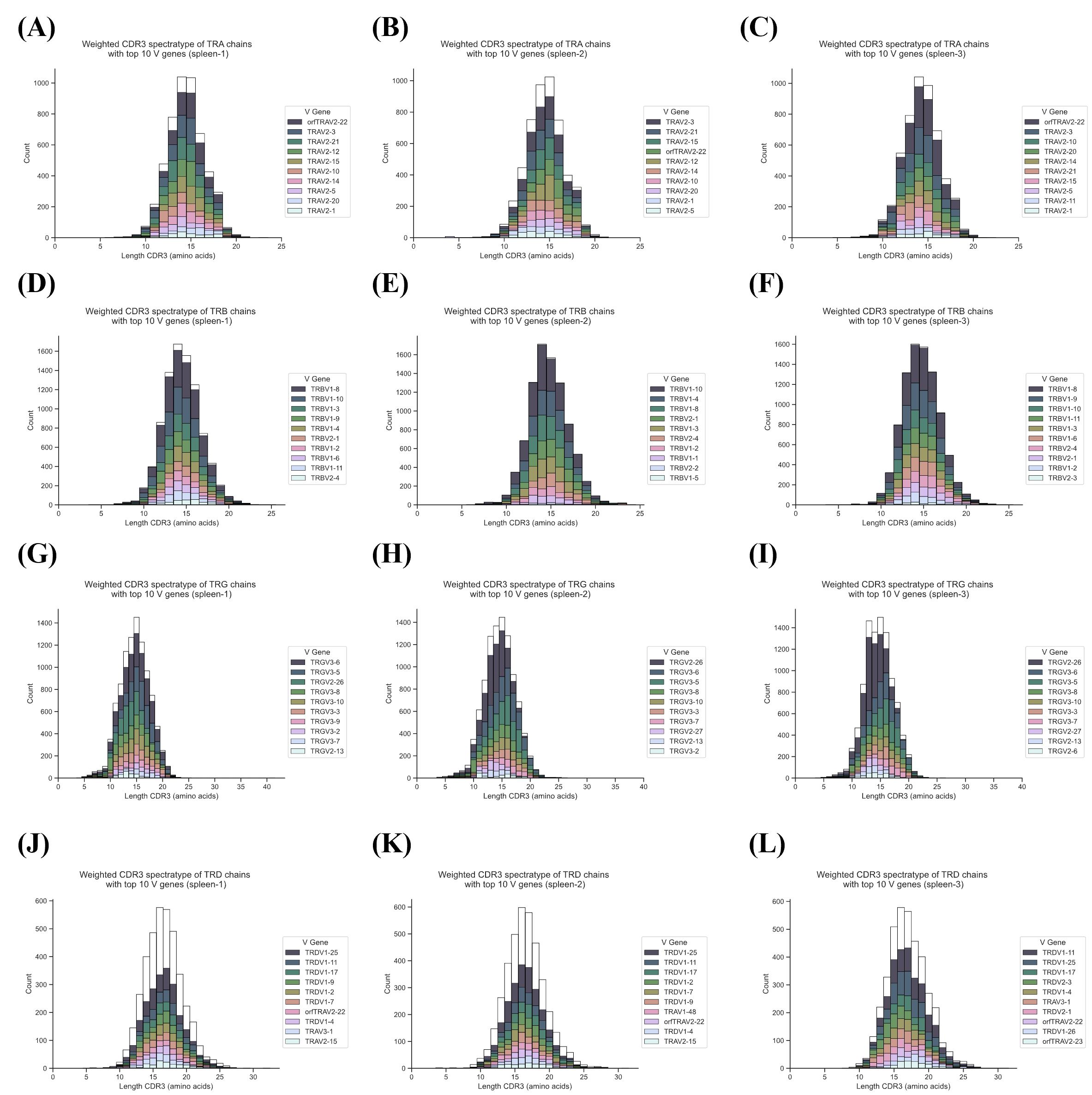


**Supplementary Figure 8.** CDR3 spectratypes with abundance of top 10 V genes.

CDR3 spectratypes showing the distribution of CDR3 amino acids lengths with frequency-weighted counts, separately for each sample. Color-coded proportions highlighting the top 10 most prevalent V genes were added. **(A-C)** TCR α spectratypes of spleens 1 - 3, **(D-F)** TCR β spectratypes of spleens 1 - 3, **(G-I)** TCR γ spectratypes of spleens 1 - 3, **(J-L)** TCR δ spectratypes of spleens 1 - 3.


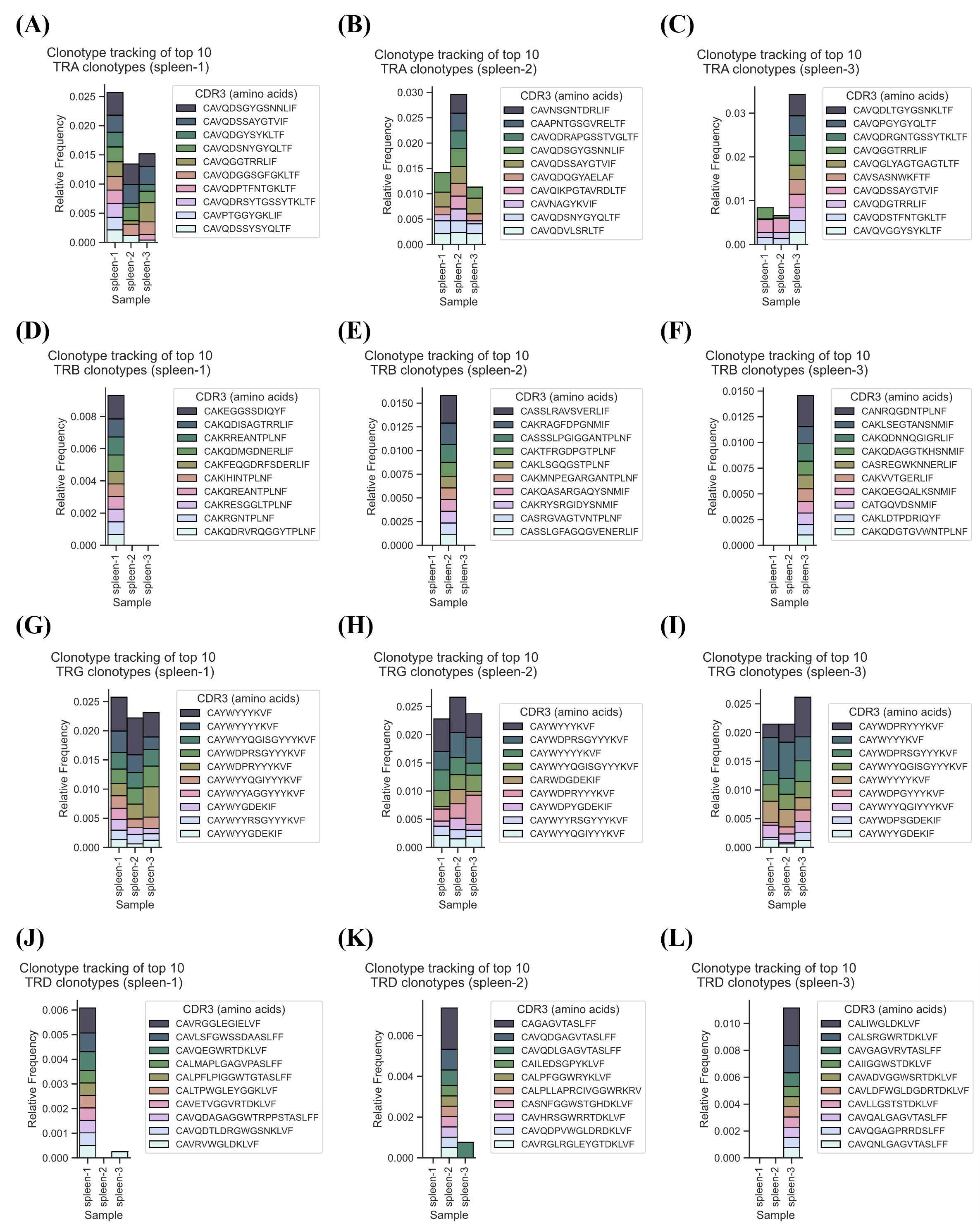


**Supplementary Figure 9.** Clonotype tracking of top 10 clonotypes across samples.

The relative frequencies of the top 10 most prevalent clonotypes in a particular sample compared across all three samples. **(A-C)** Top 10 TCR α clonotypes of spleens 1 - 3, **(D-F)** Top 10 TCR β clonotypes of spleens 1 - 3, **(G-I)** Top 10 TCR γ clonotypes of spleens 1 - 3, **(J-L)** Top 10 TCR δ clonotypes of spleens 1 - 3.

**
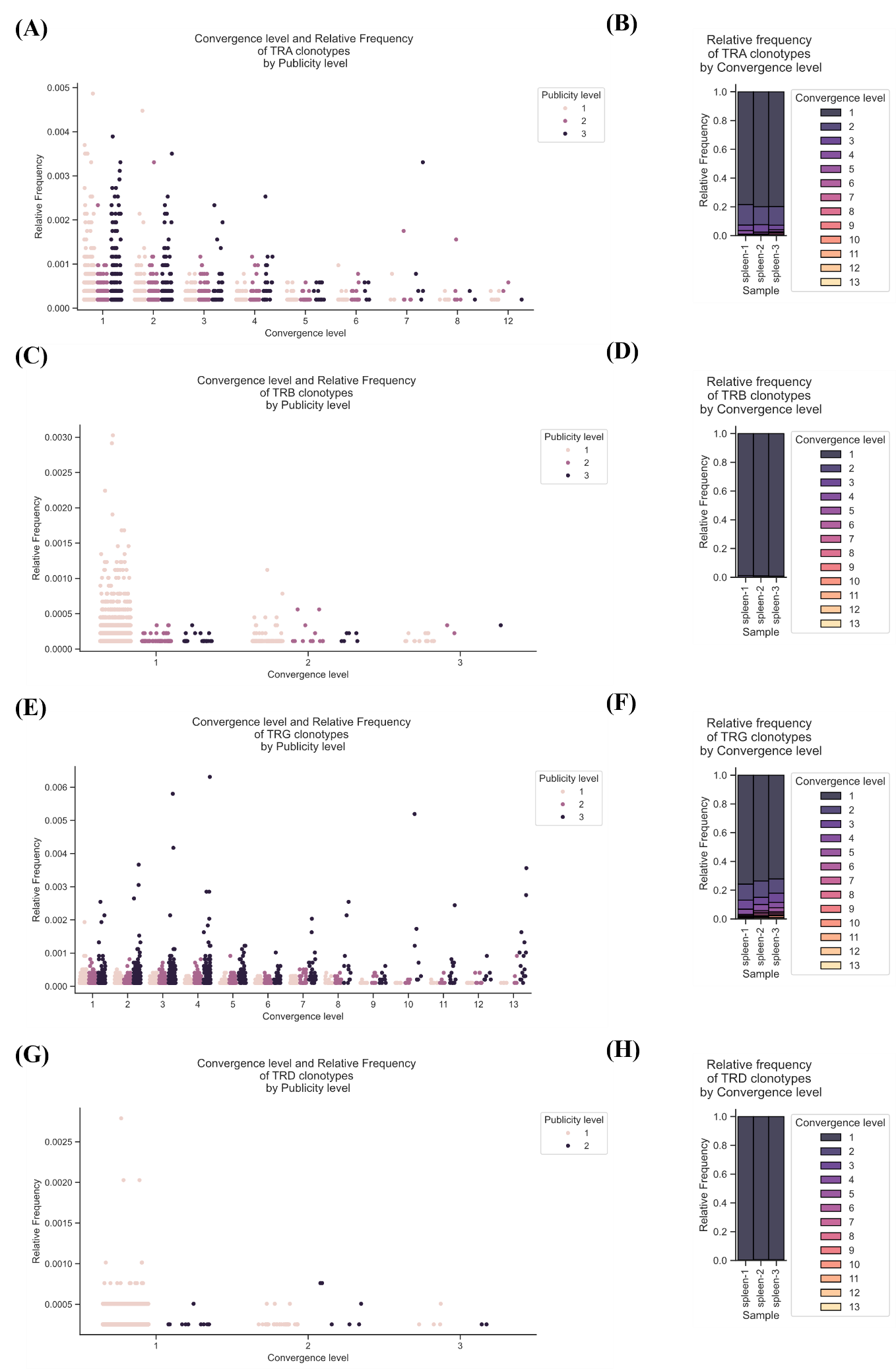
**

**Supplementary Figure 10.** Convergence of CDR3 sequences.

**(A, C, E, G)** The relative frequencies of clonotypes grouped by the number of nucleic acid sequences converging to the same amino acid CDR3 sequence (convergence level) colored by publicity level (the number of samples that share the clonotype). **(B, D, F, H)** Proportions of the TCR repertoires occupied by converging clonotypes colored by convergence level. **(A and B)** TCR α clonotypes, **(C and D)** TCR β clonotypes, **(E and F)** TCR γ clonotypes, **(G and H)** TCR δ clonotypes.

**
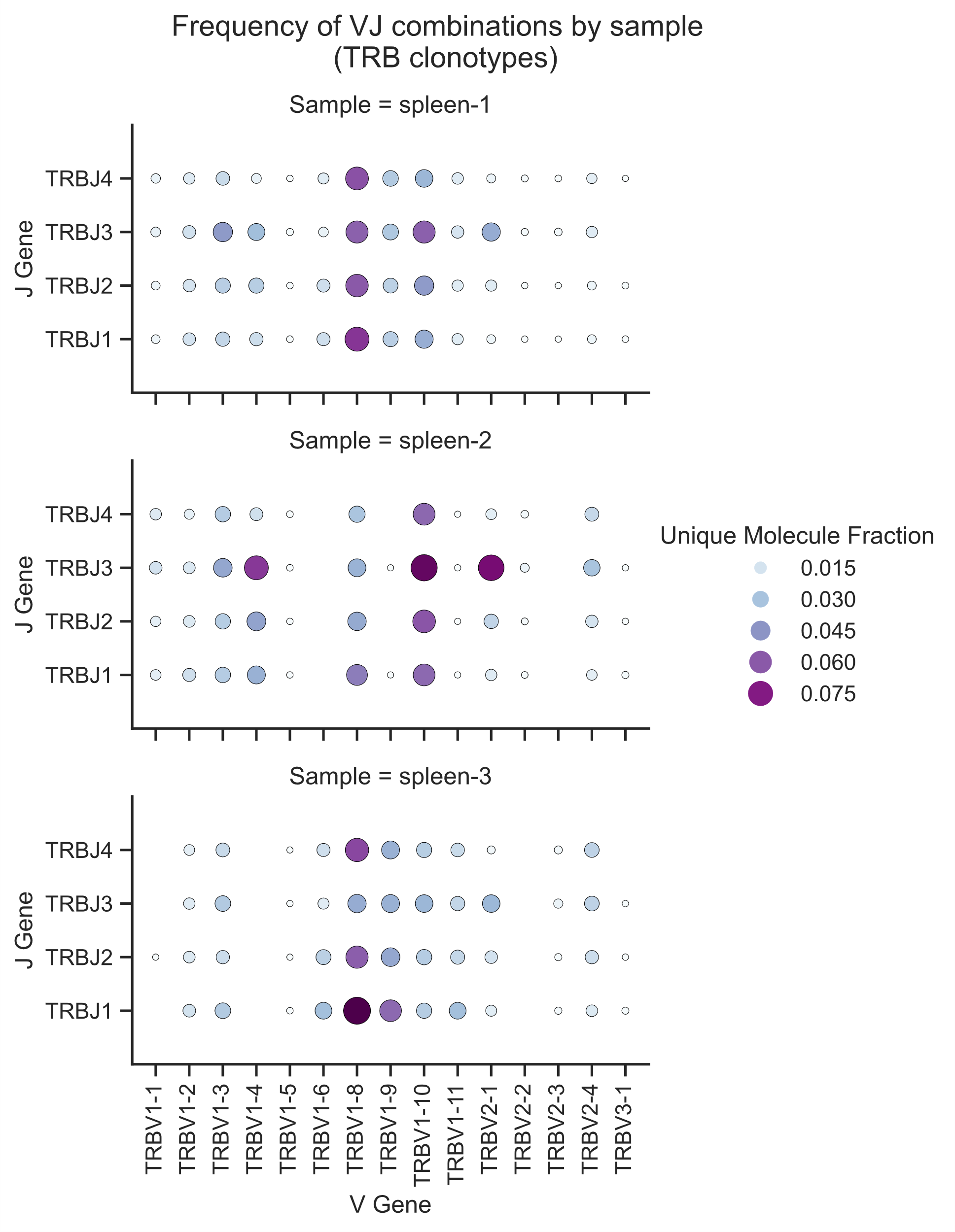
**

**Supplementary Figure 11.** Combinations of V and J genes in TCR β clonotypes depicted separately for each sample.

Bubble grid plot illustrating relative frequencies of V-J pairings, indicated by bubble size and color-coding.


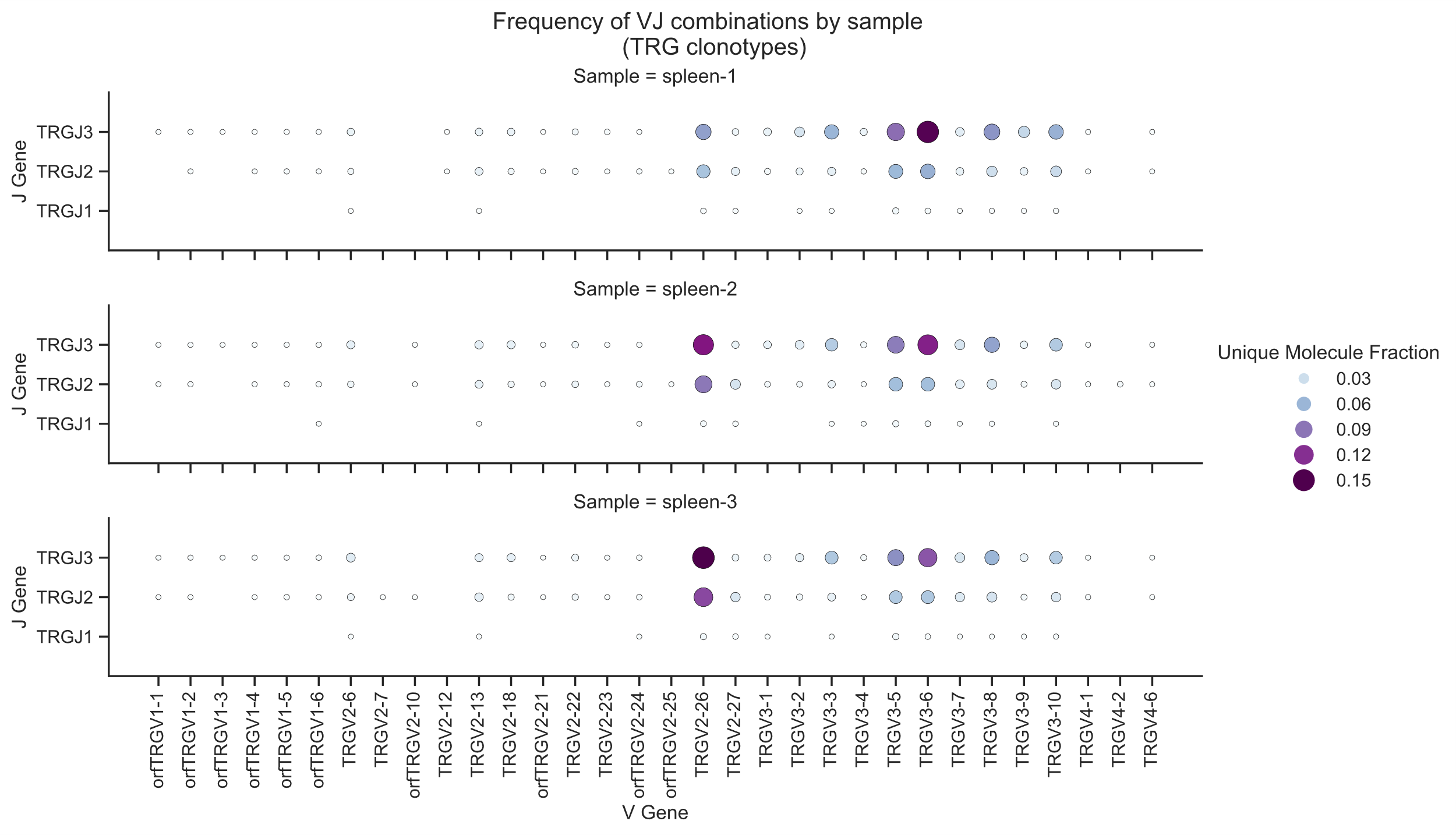


**Supplementary Figure 12.** Combinations of V and J genes in TCR γ clonotypes depicted separately for each sample.

Bubble grid plot illustrating relative frequencies of V-J pairings, indicated by bubble size and color-coding.


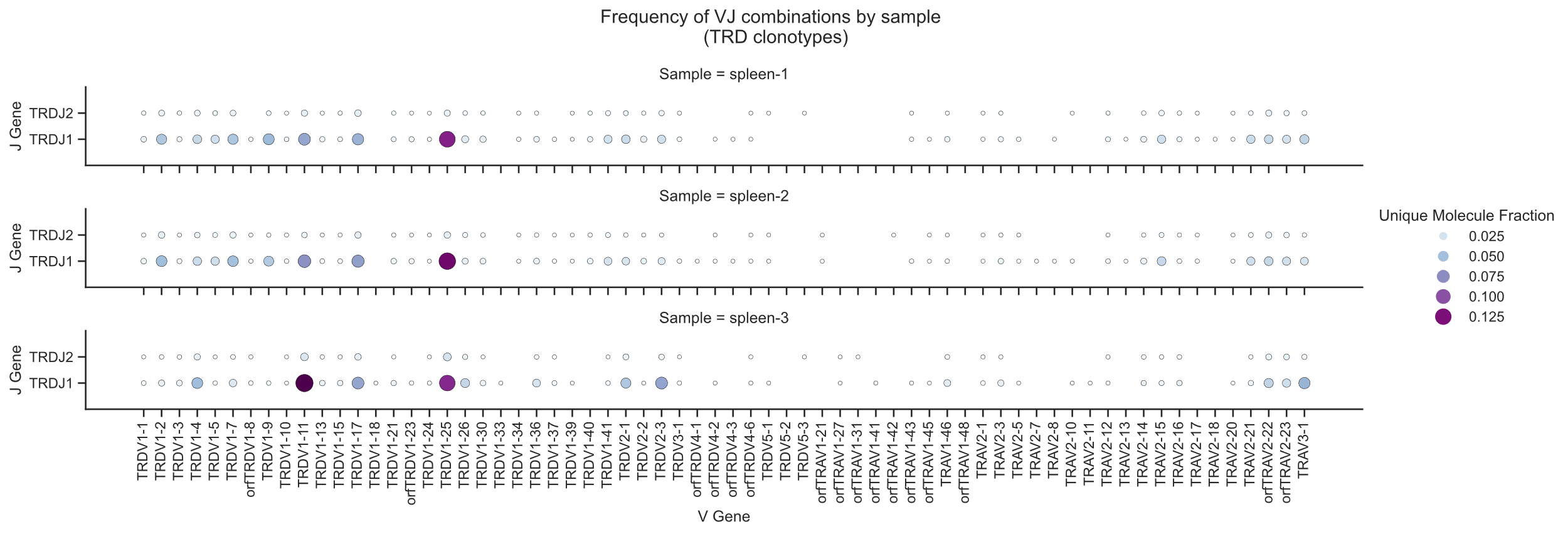


**Supplementary Figure 13.** Combinations of V and J genes in TCR δ clonotypes depicted separately for each sample.

Bubble grid plot illustrating relative frequencies of V-J pairings, indicated by bubble size and color-coding.

## Supplementary Tables

**Supplementary Table 1. Summary of TCR V(D)J genes identified.**

| **Locus** | **Chromosome** | **Gene family** | **Number / Identified by VJ-gene-finder** | | | **Total** |
| --- | --- | --- | --- | --- | --- | --- |
|  |  |  | F | ORF | P |  |
| TRA | 27 | TRAV1 | 13 / 13 | 23 / 23 | 12 | **48** |
| TRA | 27 | TRAV2 | 21 / 21 | 2 / 2 | 0 | **23** |
| TRA | 27 | TRAV3 | 1 / 1 | 0 | 0 | **1** |
| TRA | 27 | TRAJ | 57 / 56 ^(a)^ | 7 / 1 ^(b)^ | 0 | **64** |
| TRA | 27 | TRAC | 1 | 0 | 0 | **1** |
| TRB | 1 | TRBV1 | 11 / 11 | 0 | 0 | **11** |
| TRB | 1 | TRBV2 | 4 / 4 | 0 | 0 | **4** |
| TRB | 1 | TRBV3 | 1 / 1 | 0 | 0 | **1** |
| TRB | 1 | TRBD | 1 | 0 | 0 | **1** |
| TRB | 1 | TRBJ | 4 / 4 | 0 | 0 | **4** |
| TRB | 1 | TRBC | 1 | 0 | 0 | **1** |
| TRG | 2 | TRGV1 | 0 | 6 / 4 ^(c)^ | 2 | **8** |
| TRG | 2 | TRGV2 | 10 / 10 | 9 / 7 ^(c)^ | 8 | **27** |
| TRG | 2 | TRGV3 | 10 / 10 | 0 | 0 | **10** |
| TRG | 2 | TRGV4 | 3 / 3 | 0 | 5 | **8** |
| TRG | 2 | TRGJ | 3 / 3 | 0 | 0 | **3** |
| TRG | 2 | TRGC | 1 | 0 | 0 | **1** |
| TRD | 27 | TRDV1 | 35 / 35 | 3 / 2 ^(d)^ | 3 | **41** |
| TRD | 27 | TRDV2 | 3 / 3 | 0 | 0 | **3** |
| TRD | 27 | TRDV3 | 1 / 1 | 0 | 0 | **1** |
| TRD | 27 | TRDV4 | 0 | 9 / 9 | 0 | **9** |
| TRD | 27 | TRDV5 | 3 / 3 | 0 | 0 | **3** |
| TRD | 27 | TRDD | 2 | 0 | 0 | **2** |
| TRD | 27 | TRDJ | 2 / 2 | 0 | 0 | **2** |
| TRD | 27 | TRDC | 1 | 0 | 0 | **1** |
| TRD-like | 10 | TRDV | 0 | 1 / 1 | 0 | **1** |
| TRD-like | 10 | TRDD | 1 | 0 | 0 | **1** |
| TRD-like | 10 | TRDJ | 1 / 1 | 0 | 0 | **1** |
| TRD-like | 10 | TRDC | 1 | 0 | 0 | **1** |

*Note.* Reason why specific genes were not identified by *VJ-gene-finder:*

^(a^) Unusual splice donor; ^(b)^ no “W/FG.G” motif; ^(c)^ “2^nd^ Cys” missing; ^(d)^ unusual motif at “2^nd^ Cys”

**Supplementary Table 2. Sequence comparison with previously published TCRβ V(D)J genes.**

| **Query Name*** | **Match Name^§^** | **Percent Identity** | **Mismatches** |
| --- | --- | --- | --- |
| V1.11 | TRBV1-1*01 | 99.65% | 1 |
| V1.10 | TRBV1-9*01 \| TRBV1-8*01 | 99.30% | 2 |
| V1.9 | TRBV1-10*01 | 98.95% | 3 |
| V1.8 | TRBV1-8*01 | 99.30% | 2 |
| V1.7 | TRBV1-6*01 | 97.90% | 6 |
| V1.6 | TRBV1-11*01 \| TRBV1-10*01 \| TRBV1-7*01 | 97.90% | 6 |
| V1.5 | TRBV1-8*01 | 98.95% | 3 |
| V1.4 | TRBV1-6*01 | 97.90% | 6 |
| V1.3 | TRBV1-6*01 | 98.25% | 5 |
| V1.2 | TRBV1-1*01 | 97.90% | 6 |
| V1.1 | TRBV1-2*01 | 97.55% | 7 |
| V2.3 | TRBV2-1*01 | 98.92% | 3 |
| V2.2 | TRBV2-2*01 | 98.92% | 3 |
| V3.1 | TRBV3-1*01 | 100.00% | 0 |
| V2.1 | TRBV2-1*01 | 95.32% | 13 |
| V2.4 | TRBV2-4*01 | 98.56% | 4 |
| D | TRBD1*01 | 100.00% | 0 |
| J1 | TRBJ1*01 | 100.00% | 0 |
| J2 | TRBJ2*01 | 100.00% | 0 |
| J3 | TRBJ3*01 | 100.00% | 0 |

*Note.* * Zhang et al. (2020): https://doi.org/10.1016/j.vetimm.2019.109974; **^§^** this study;

**Supplementary Table 3. Sequence comparison with previously published TCRγ V(D)J genes.**

| **Query Name*** | **Match Name^§^** | **Percent Identity** | **Mismatches** |
| --- | --- | --- | --- |
| V1.1ORF | pTRGV1-8*01 | 95.96% | 12 |
| V1.2 | orfTRGV1-5*01 | 98.04% | 6 |
| V1.3 | orfTRGV1-6*01 | 100.00% | 0 |
| pV1.4 | orfTRGV1-6*01 | 100.00% | 0 |
| V1.5ORF | pTRGV1-7*01 | 94.95% | 15 |
| V1.6 | orfTRGV1-1*01 | 97.39% | 8 |
| V2.1 | TRGV2-27*01 | 99.67% | 1 |
| V2.2 | TRGV2-26*01 | 100.00% | 0 |
| V2.3 | orfTRGV2-25*01 \| orfTRGV2-16*01 \| orfTRGV2-10*01 | 99.35% | 2 |
| V2.4 | orfTRGV2-24*01 \| orfTRGV2-9*01 | 100.00% | 0 |
| V2.5 | TRGV2-18*01 | 99.67% | 1 |
| V2.6 | orfTRGV2-25*01 \| orfTRGV2-16*01 \| orfTRGV2-10*01 | 99.02% | 3 |
| pV2.7 | orfTRGV2-24*01 \| orfTRGV2-9*01 | 100.00% | 0 |
| V2.8 | TRGV2-13*01 \| TRGV2-7*01 | 100.00% | 0 |
| pV2.9 | TRGV2-26*01 | 99.35% | 2 |
| pV2.10 | orfTRGV2-4*01 | 97.37% | 8 |
| pV2.11 | orfTRGV2-24*01 \| orfTRGV2-9*01 | 100.00% | 0 |
| V2.12 | TRGV2-13*01 \| TRGV2-7*01 | 100.00% | 0 |
| V2.13 | TRGV2-26*01 | 99.67% | 1 |
| V2.14 | orfTRGV2-21*01 | 99.35% | 2 |
| V2.15 | orfTRGV2-24*01 \| orfTRGV2-9*01 | 100.00% | 0 |
| V2.16 | TRGV2-23*01 \| TRGV2-13*01 \| TRGV2-7*01 | 99.35% | 2 |
| V2.17 | orfTRGV2-21*01 | 96.41% | 11 |
| V2.18ORF | orfTRGV2-24*01 \| orfTRGV2-9*01 \| orfTRGV2-3*01 | 97.06% | 9 |
| V2.19 | TRGV2-18*01 \| TRGV2-12*01 | 97.71% | 7 |
| V3.1 | TRGV3-10*01 | 99.32% | 2 |
| V3.2 | TRGV3-5*01 | 99.66% | 1 |
| V3.3 | TRGV3-4*01 | 97.61% | 7 |
| V3.4 | TRGV3-7*01 | 99.32% | 2 |
| pV3.5 | TRGV3-8*01 \| TRGV3-6*01 | 96.59% | 10 |
| V3.6 | TRGV3-7*01 | 99.32% | 2 |
| V3.7 | TRGV3-8*01 | 98.63% | 4 |
| V3.8 | TRGV3-2*01 | 97.95% | 6 |
| V3.9 | TRGV3-7*01 \| TRGV3-4*01 | 96.59% | 10 |
| V4.1 | TRGV4-6*01 | 98.27% | 5 |
| V4.2 | TRGV4-6*01 | 98.96% | 3 |
| V4.3 | TRGV4-6*01 | 98.96% | 3 |
| pV4.4 | TRGV4-2*01 \| TRGV4-1*01 | 87.54% | 36 |
| pV5.1 | pTRGV2-8*01 \| pTRGV2-2*01 | 70.63% | 74 |
| pV5.2 | pTRGV2-8*01 \| pTRGV2-2*01 | 70.63% | 74 |
| pV5.3 | pTRGV2-8*01 \| pTRGV2-2*01 | 71.03% | 73 |
| pV6.1 | pTRGV4-4*01 | 73.70% | 76 |
| pV6.2 | pTRGV4-4*01 \| TRGV4-2*01 \| TRGV4-1*01 | 75.43% | 71 |
| pV6.3 | pTRGV4-4*01 | 73.50% | 75 |
| J1 | TRGJ1*01 | 100.00% | 0 |
| J2 | TRGJ2*01 | 100.00% | 0 |
| J3 | TRGJ3*01 | 100.00% | 0 |

*Note.* * Zhang et al. (2021): https://doi.org/10.1186/s12864-021-07975-7; **^§^** this study;
